# A Scoping Review of Machine Learning Applications Across Epidemiological Stages of Zoonotic Disease

**DOI:** 10.1155/tbed/2215823

**Published:** 2026-05-14

**Authors:** Yinsheng Zhang, Yifan Sun, Jinchen Wang, Luqi Wang, Ruying Fang, Xiaolong Wu, Xin Yang, Yiyang Guo, Sen Li

**Affiliations:** ^1^ School of Environmental Science and Engineering, Huazhong University of Science and Technology, Wuhan, 430074, Hubei, China, hust.edu.cn; ^2^ Institute of Artificial Intelligence, Huazhong University of Science and Technology, Wuhan, 430074, Hubei, China, hust.edu.cn; ^3^ School of Artificial Intelligence and Automation, Huazhong University of Science and Technology, Wuhan, 430074, Hubei, China, hust.edu.cn; ^4^ Earth and Life Institute, Université Catholique de Louvain (UCLouvain), Louvain-la-Neuve, B1348, Belgium, uclouvain.be

**Keywords:** animal–human interface, epidemiological stages, machine learning, zoonotic diseases

## Abstract

Emerging zoonotic diseases represent a significant threat to global health. While machine learning (ML) holds promise for their management, a comprehensive understanding of how these technologies are applied across the entire animal‐to‐human transmission pathway is lacking. This scoping review systematically maps ML applications in zoonotic disease management to identify research trends, methodological approaches, and critical gaps across different epidemiological stages and functional domains. We organize the literature along two dimensions: epidemiological stages, from animal hosts to human populations, and functional domain, including diagnosis, epidemiology, and intervention. We searched PubMed and Web of Science for studies on 14 preselected high‐priority zoonotic diseases. The search string combined keywords for the selected diseases, ML techniques, and functional applications (diagnosis, epidemiology, and intervention). A total of 966 studies were included in the final analysis, of which 72.8% focused on COVID‐19. Our analysis shows robust ML performance in clinical diagnostics, epidemic forecasting, and intervention optimization within human populations. However, critical gaps persist, only 1.96% of studies examined the animal–human interface, no ML models explicitly targeted spillover prevention, and studies on animal‐reservoir surveillance remain limited. All spillover studies originated from high‐income or upper‐middle‐income countries (UMICs), in contrast with low‐ and lower‐middle‐income countries (LMICs) contributing 21.4% of human‐stage studies. These findings reveal a pronounced mismatch between research investment and spillover risk and highlight the need for greater emphasis on spillover mechanisms, enhanced integration of cross‐species transmission dynamics, and methods suitable for surveillance in resource‐limited settings. Addressing these imbalances is essential for advancing a shift from reactive outbreak response to proactive spillover prevention within a One Health framework.

## 1. Introduction

Emerging and re‐emerging zoonotic diseases, caused by pathogens that naturally spread between animals and humans, pose significant challenges to global health, economies, and food security [1, 2]. Historic pandemics, such as the Black Death, Spanish Flu, and most recently COVID‐19, as well as ongoing epidemics such as human immunodeficiency virus (HIV)/AIDS and recurrent Ebola outbreaks, illustrate their devastating impacts [3]. More than 60% of emerging infectious diseases originate from animal sources, and about 75% of new human pathogens have zoonotic origins. Understanding and preventing cross‐species transmission are therefore critical objectives for pandemic surveillance programs [4, 5].

The distinctive features of zoonotic diseases, including high emergence frequency, broad societal impacts, and potential for rapid global spread, render reactive outbreak responses inadequate. The COVID‐19 pandemic showed how a localized spillover event can develop into a global crisis within weeks. These characteristics highlight the need for proactive, multilevel surveillance and prediction systems that enable intervention at each stage of the transmission pathway, from animal hosts to human populations. Therefore, a central challenge is developing analytical tools that correspond to the dynamic information available at each stage of pathogen emergence, enabling the design of targeted intervention strategies.

Zoonotic transmission involves complex, multihost ecologies in which pathogens circulate within wildlife or domestic animals, occasionally spillover to humans, and may adapt to sustain human‐to‐human transmission [6]. This process is influenced by host biodiversity, ecological change, vector dynamics, social behaviors, and environmental factors [7]. Mathematical modeling is essential for analyzing this complexity and developing effective intervention policies and research agendas [8]. These analytic tools include process‐based mechanistic models (e.g., compartmental and agent‐based models) or statistical correlation analyses [9, 10]. However, traditional modeling methods face limitations when applied to the intricate ecology of zoonoses. Process‐based socioecological models, for instance, often encounter a trade‐off between excessive complexity that prevents large‐scale validation and oversimplification that fails to capture the nonlinear, interactive dynamics of zoonotic disease transmission [11]. These limitations highlight the need for innovative approaches that can bridge the gap between mechanistic understanding and practical predictive ability.

Recent advances in artificial intelligence (AI), especially in machine learning (ML), provide valuable tools to address these limitations [12]. ML algorithms excel at identifying hidden patterns and nonlinear relationships within the complex characteristics of zoonotic systems [13]. This data‐driven capability enables more accurate disease modeling dynamics without relying on a priori mechanistic assumptions [14]. Moreover, the adaptive learning ability of ML models makes them particularly well‐suited for addressing the dynamic nature of zoonotic infections. These capabilities can be leveraged at different stages of the transmission pathway, from reservoir surveillance and spillover risk mapping to forecasting human outbreaks and optimizing interventions.

Existing reviews have synthesized aspects of ML applications in zoonotic disease research [15–17]; however, most focus on specific epidemiological stages. Many reviews concentrate on posthuman infection stages, such as human diagnostics, therapeutic development, or risk assessment for human‐to‐human transmission [18]. This narrow scope limits the ability to understand and predict earlier stages where interventions may be most effective [19]. Moreover, a critical but underexplored area concerns how ML has been utilized across the complete pathogen transmission pathway. This lack of comprehensive synthesis makes it difficult to identify which stages have benefited from innovative ML applications that are worth consolidating and which remain underdeveloped. An integrated perspective is increasingly essential in a world shaped by climate change, habitat encroachment, and increased global mobility, as effective prevention requires coordinated approaches enabling surveillance and intervention across epidemiological stages, from animal reservoirs to human populations.

In this study, we conduct a scoping review to investigate ML applications in zoonotic disease management. Our objectives are to (1) systematically map ML applications across key epidemiological stages (animal hosts and vectors, animal–human interface, and to human populations) of zoonotic pathogens; (2) identify the predominant ML algorithms and data types employed for diagnosis, epidemiology, and intervention within each context; and (3) analyze distribution of research focus, methodological trends, and critical bottlenecks in current research. The review follows the natural trajectory of zoonotic transmission, providing a narrative synthesis of findings at each epidemiological stage. By integrating insights from ecology, epidemiology, data science, and public health, this review offers an interdisciplinary roadmap for developing effective strategies and advancing pandemic‐prevention efforts targeting zoonotic diseases.

## 2. Materials and Methods

### 2.1. Conceptual Framework

The analytical structure of this review is organized along the zoonotic disease transmission pathway, providing a framework that traces pathogen emergence from origin to societal impact. This pathway comprises three key epidemiological stages: (i) surveillance at the source, involving early monitoring of pathogens at animal reservoirs and vectors; (ii) the animal–human interface, focusing on the assessment of spillover risk; and (iii) management in human populations, addressing the clinical and public health responses following established human‐to‐human transmission.

To ensure analytical consistency across these stages, we classified each study according to its primary functional objective within one of three domains. “Diagnosis” refers to the methods and processes used to determine the status of an individual unit of concern, such as a patient, animal host, vector, or pathogen. This domain includes both clinical diagnostics (e.g., assessing a patient’s disease state and prognosis) and source identification (e.g., detecting pathogens in animals or characterizing a virus’s zoonotic potential). “Epidemiology” involves the analysis, modeling, and forecasting of disease transmission dynamics across populations, space, and time. It seeks to elucidate risk patterns, identify key drivers (e.g., environmental, social, or network‐based factors), and predict future epidemic trajectories. “Intervention” comprises population‐level models designed to inform public health action, using evaluative frameworks to assess prior measures or prescriptive frameworks to identify optimal future control policies. This framework systematically reveals the scientific questions and corresponding ML methods for each application domain and epidemiological stage.

### 2.2. Disease Selection

Zoonoses, as defined by the World Health Organization, are “diseases and infections that are naturally transmitted between vertebrate animals and humans [20]”. We prioritized diseases recognized for their significant global health burden, high mortality rates, or pandemic potential, which require urgent research and development action [21]. Moreover, to ensure our findings were broadly relevant across the spectrum of zoonotic challenges, we chose diseases that exemplify a variety of transmission routes. This includes vector‐borne (e.g., malaria and Zika virus), direct contact (e.g., Ebola virus and Nipah virus), and airborne/droplet transmission (e.g., COVID‐19 and highly pathogenic avian influenza [HPAI]). The final list of selected diseases is HPAI, malaria, Nipah virus, MERS‐CoV, Ebola virus, Zika virus, West Nile fever, chikungunya, Rift Valley fever (RVF), H1N1 influenza, monkeypox, COVID‐19, HIV, and Lassa fever. A detailed summary of each disease and its characteristics is provided in Table S1.

### 2.3. Literature Search Strategy

This scoping review synthesizes the literature on the application of AI in managing zoonotic diseases, adhering to the PRISMA‐ScR guidelines [22]. Given the expansive and interdisciplinary scope of this topic, our search strategy was designed to balance comprehensiveness with feasibility. We developed the search strategy based on the conceptual framework and exploratory searches of the literature. The final search string, detailed in Text S1, was designed to be comprehensive within a feasible scope and was applied systematically across the selected databases on July 10, 2023. This review focuses on three key questions: (i) How have ML techniques been applied across key epidemiological stages of zoonotic diseases? (ii) Which ML algorithms and data types are most commonly employed in zoonotic disease management? (iii) What distributional patterns and research gaps exist in ML applications across different diseases, geographical regions, and application domains? We searched PubMed and Web of Science using a three‐component search string (Test S1). This string combined keywords for (a) specific zoonotic diseases, (b) AI and ML, and (c) primary functional applications using Boolean operators (“AND” and “OR”).

### 2.4. Selection Criteria and Procedure

All retrieved records were imported into EndNote X9.3.3 to remove duplicates before screening. Three reviewers (YZ, YS, and JW) independently screened all titles and abstracts during the primary screening stage. Full‐text screening was conducted independently by nine reviewers (YZ, YS, JW, RF, LW, XY, XW, YG, and SL). Any discrepancies at either stage were resolved through team discussion to ensure consensus. This entire selection process is illustrated in Figure S1.

Studies were included if they met the following criteria: (i) focused on one of the 14 preselected zoonotic diseases; (ii) employed at least one ML or deep learning (DL) algorithm for analysis, beyond simple data preprocessing; and (iii) addressed a question related to our defined diagnosis, epidemiology, or intervention. Eligible AI techniques include all ML and DL models, excluding statistical models and fuzzy logic systems. Conversely, studies were excluded if they were (i) nonoriginal research (e.g., reviews, commentaries, editorials, and posters) or nonpeer‐reviewed articles; (ii) not published in English; (iii) used ML/DL algorithms only for data preprocessing, or described algorithms without reporting training and validation/testing steps; (iv) employed only deterministic models (e.g., ordinary differential equations), traditional statistical models, or fuzzy logic systems without an ML component; (v) focused on topics outside the review’s scope, such as fundamental molecular biology, drug discovery, vaccine development, or psychology; or (vi) lacked sufficient methodological detail, did not report numerical results, or the full text was inaccessible.

The boundaries between epidemiological stages sometimes can be ambiguous, particularly for studies such as ecological niche models and host prediction studies that may inform multiple stages depending on their analysis framing. To ensure transparent and reproducible classification, each included study was assigned to one or more epidemiological stages based on three operational criteria applied in combination: (i) the prediction target of the model, (ii) whether the analysis explicitly linked the animal and human systems, and (iii) how the research question was framed by the original papers. Specifically, studies were assigned to the animal reservoir/vector stage when the prediction target remained within the animal or vector system, and the research question did not reference cross‐species transmission. This stage includes, for example, detecting pathogens in known host populations, classifying vector species, modeling vector distribution and abundance, and predicting disease outbreaks within animal populations. Studies were assigned to the animal–human interface when the analysis explicitly bridged the animal and human systems. This includes studies aimed at discovering novel host or vector species that may serve as sources of human infection, assessing a pathogen’s potential to infect humans, attributing human infections to specific animal lineages, or modeling the probability and determinants of spillover events using human infection data or human–animal contact data as the outcome. Studies were assigned to the human population stage when both the input data and the research question concerned postspillover dynamics, such as clinical diagnosis, human epidemic forecasting, or intervention evaluation. When a study contained research objectives relevant to more than one stage, it was assigned to each applicable stage. Representative examples of borderline classification decisions are provided in Table S3.

### 2.5. Data Analysis

Two reviewers (YZ and YS) independently extracted data from all included studies using a standardized form, with any disagreements resolved through consensus. The extracted data variables included (a) bibliographic information (first author and year of publication); (b) study characteristics (country of study and zoonotic disease); (c) epidemiological stage (animal reservoir/vector, human–animal interface, or human population); (d) functional domain (diagnosis, epidemiology, or intervention); (e) ML model details (model type, specific algorithm, and model task); (f) data characteristics (data types and specific dataset); (g) data characteristics (data types and specific dataset); and (h) transmission mode (vector‐borne, direct contact, or airborne/droplet, following the classification in Section 2.2) and country income level (high‐income countries [HICs], upper‐middle‐income countries [UMICs], or low‐ and lower‐middle‐income countries [LMICs], according to the World Bank Atlas method). Given the heterogeneity in methodologies and outcomes, a formal meta‐analysis was deemed inappropriate. Instead, we performed a narrative synthesis of the extracted data, grouping the findings according to our conceptual framework to identify key trends, patterns, and gaps.

## 3. Results

### 3.1. Study Selection and Characteristics

**Figure 1:**
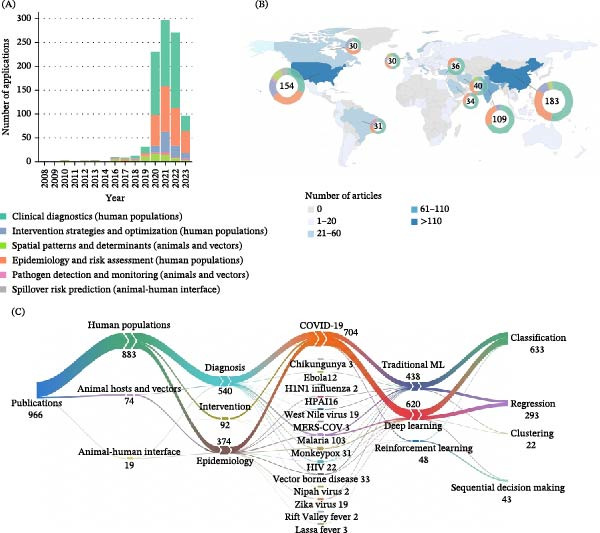
Global distribution and research focus of zoonoses publications. (A) Temporal trends of ML applications from 2008 to 2023, with bars color‐coded by research focus categories. (B) Geographical distribution of the reviewed publications, where countries are color‐coded by publication count. (C) Sankey diagram illustrating the connections among research categories, such as epidemiological stages, disease focus, and techniques. The numbers indicate the publication count within each category. Abbreviations: HIV, human immunodeficiency virus; HPAI, highly pathogenic avian influenza; Traditional ML, traditional machine learning.

**Figure 2:**
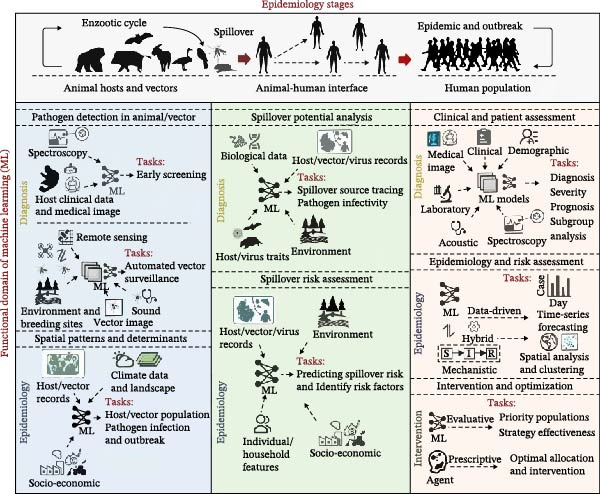
An illustrative summary of ML applications for zoonotic disease management, based on the reviewed articles. The framework summarizes the included articles based on the framework constructed from the epidemiological stage (horizontal axis) and the functional domain of ML (vertical axis). Each panel within the framework details a specific application area, illustrating the typical data inputs, the central role of ML models, and the corresponding research tasks.

Our systematic search yielded 27,390 papers, from which 14,185 unique articles remained after the removal of duplicates. Subsequent screening of titles, keywords, and abstracts identified 3662 potentially relevant papers. Following a full‐text evaluation, 966 articles met the final eligibility criteria (Figure S1). To systematically organize our analysis, we constructed a conceptual framework based on two dimensions: the epidemiological stage of the zoonotic transmission pathway and the functional domain of the ML application (see Section 2). Within this framework, we identified the typical data inputs, algorithms, and research tasks for the main ML applications. We grouped these application areas into six primary categories: clinical diagnostics and patient assessment (diagnostic modeling within human population, *n* = 515), zoonotic epidemiology and risk assessment (epidemiological modeling within human population, *n* = 315), intervention strategies and optimization (interventional modeling within human population, *n* = 92), pathogen detection in animal reservoirs and vector monitoring (diagnostic modeling within animals, *n* = 18), spatial patterns and determinants of zoonotic diseases in animals and disease vectors (epidemiological modeling in animals, *n* = 56), and spillover risk prediction at the animal–human interface (*n* = 19) (Figure 1A). The following sections provide a detailed description of each application area identified within this framework (Figure 2). Data types used in zoonotic ML modeling were classified based on their characteristics and acquisition methods, including demographic, epidemiological, clinical, laboratory, environmental, biological, image/video, medical image, entomological, acoustic, socioeconomic, spectroscopy, and text data (Table 1). These categories are not mutually exclusive, as a single study can encompass multiple application areas and data types (Table S2).

**Table 1:** Description of data types used in zoonotic ML modeling in the reviewed articles.

Data type	Description	Examples
Demographic data	Data describing the characteristics of individual human subjects or households	Patient age, gender, ethnicity, individual comorbidities, household size, and family income
Clinical data	Information on a human patient’s health status obtained through direct observation or basic measurement	Symptoms (fever, headache, and cough), vital signs (blood pressure and heart rate), medical history, and patient‐reported outcomes
Laboratory data	Quantitative results from the analysis of individual biological samples in a laboratory setting	Complete blood count (CBC), polymerase chain reaction (PCR) test results, and inflammatory markers (e.g., C‐reactive protein)
Medical images	Images acquired through medical or veterinary procedures for individual‐level diagnosis or assessment	Chest X‐ray images, blood smear images, and CT scans
Epidemiological data	Data on the incidence, distribution, and dynamics of diseases at a population level	Confirmed cases, recovered cases, and death rates
Socioeconomic data	Aggregated data describing the societal context, including economic, behavioral, and structural characteristics of a population	Population density, regional GDP, educational attainment, human mobility patterns, air travel volumes, and access to healthcare
Environmental data	Structured data describing the climate, geography, topography, and land cover, often derived from remote sensing	Temperature, precipitation, elevation, land cover type (e.g., forest), vegetation indices (e.g., NDVI), and road density
Biological data	Data describing organisms at the molecular, genetic, or protein level	Genome/protein sequences, species‐level traits, vector‐virus traits, and seroprevalence data
Entomological data	Data specifically related to insect vectors, which can include both population‐level metrics and individual records or images	Vector abundance, pupation rates, and vector presence/absence data
Acoustic data	Sound recordings from an individual subject (human or animal) used for diagnostic screening or analysis	Cough recordings, breath sounds, and pulmonary auscultation data
Images or videos	Unstructured imagery or video footage not from a medical context, often used for environmental analysis and direct input into computer vision models	Aerial/drone images of potential breeding sites, images from citizen science platforms (e.g., mosquito alert)
Animal data	A broad category for noninsect animals, covering multiple scales: individual‐level clinical/physiological data, species traits, and population‐level distribution and prevalence	Animal/vector occurrence, species richness, population densities, and animal movement/telemetry data
Spectroscopy	Data from spectral analysis of an individual sample used to determine its chemical or physical composition	Near‐infrared spectroscopy (NIRS) for mosquito age/species identification and mass spectrometry for detecting infection status
Text data	Unstructured textual information from various sources, which can be analyzed to infer both individual‐level (e.g., clinical notes) and population‐level (e.g., news reports) insights	Clinical notes, scientific literature, news reports, and social media posts (e.g., Reddit)

**Figure 3:**
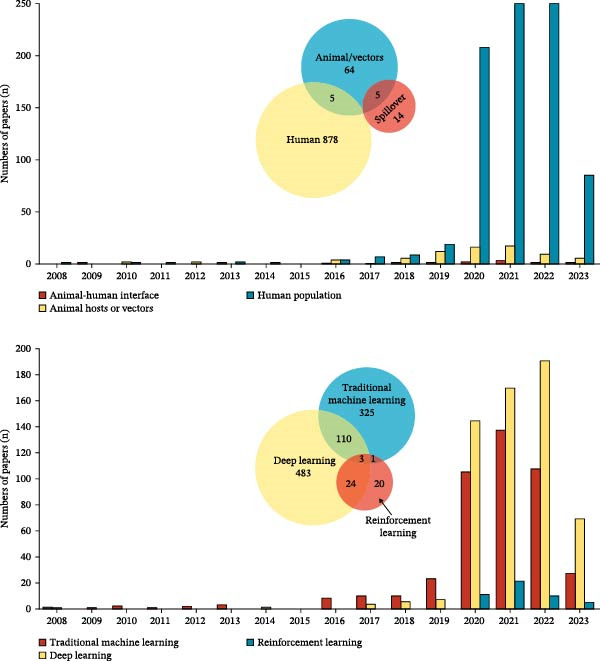
Temporal trends and overlaps in reviewed publications. The upper panel displays the temporal trend and categorical overlap of publications stratified by transmission stage; the bar chart shows the annual counts, and the Venn diagram illustrates the overlaps. Similarly, the lower panel depicts the annual distribution and overlap for different modeling techniques, including traditional machine learning, deep learning, and reinforcement learning.

The geographic distribution of publications on global zoonotic diseases was widespread (Figure 1B). China led with 183 publications, followed by the United States (154) and India (109). This distribution is likely driven by factors such as these countries’ large endemic populations, alongside substantial research funding and advanced technological infrastructure. A surge in ML development occurred between 2020 and 2022, primarily targeting pathogens in human populations (Figure 1C). COVID‐19 was the focus of the vast majority of publications (704/966, 72.8%), followed by malaria (103, 10.7%), vector‐borne diseases (32, 3.4%), and monkeypox (31, 3.2%). The COVID‐19 pandemic prompted significant research investments from global health institutions and governments, accelerating the development of ML technologies for clinical diagnosis and epidemiological prediction in humans. In contrast, studies on spillover transmission modeling were notably scarce (19, 1.96%). Temporal analysis reveals a notable increase in ML studies addressing zoonotic diseases in human populations from 2020 to 2022, coinciding with the COVID‐19 pandemic (Figure 3, top panel). DL consistently exceeded traditional ML approaches from 2020 onward, a pattern that closely tracks the rise of COVID‐19 studies and the large‐scale imaging and surveillance data they provided. For both methodologies, classification and regression were the predominant modeling tasks, whereas reinforcement learning (RL) was mainly used for sequential decision‐making (Figures 1C and 3, bottom panel).

### 3.2. Clinical Diagnostics and Patient Assessment in Human Populations

**Figure 4:**
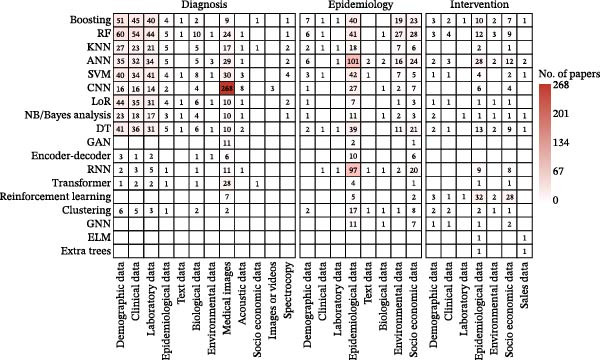
Co‐occurrence of ML models (rows) and data types (columns) applied to the management of human populations across three functional domains (diagnosis, epidemiology, and intervention). Cell values indicate the number of model‐data coapplications; a single publication may contribute to multiple cells if it utilized multiple models or data types. Color intensity reflects the number of papers. Abbreviations: ANN, artificial neural network; CNN, convolutional neural network; DT, decision tree; ELM, extreme learning machine; GAN, generative adversarial network; GNN, graph neural network; KNN, K‐nearest neighbors; LoR, logistic regression; NB, Naive Bayes; RF, random forest; RNN, recurrent neural network; SVM, support vector machine.

This domain encompasses 515 publications focused on applying ML to enhance clinical decision‐making for human diseases, utilizing 15 different ML models across 12 data types (Figure 4, diagnosis panel). The research primarily addresses three hierarchical clinical tasks: disease detection and diagnosis (*n* = 412), patient severity assessment and lesion segmentation (*n* = 68), and prognosis prediction (*n* = 56), with many studies tackling multiple objectives. The methodological landscape is dominated by traditional ML and DL (Figure S2A), which are applied to a range of primary inputs, including medical images, demographic, clinical, and laboratory data (Figure 4 and Figure S2B). A strong synergy between data modality and model choice was observed, with the most favored combinations being convolutional neural network (CNN) with medical images (*n* = 268) and random forest (RF) with demographic data (*n* = 60) (Figure 4; Table S2).

The most frequent application of ML was in disease detection and diagnosis. Within this domain, analyses based on medical imaging, covered in 324 studies, constituted the most prominent category. DL models, particularly CNN (*n* = 292), were paramount for augmenting or automating visual interpretation. Key innovations such as transfer learning (*n* = 15) were frequently employed to address data scarcity, as exemplified by the previous study [23]. In addition, many studies have used these techniques for detailed patient assessment. For instance, in the task of segmentation of infected lesions, a key component of severity evaluation, advanced architectures incorporating transformers and attention mechanisms (*n* = 32) were employed. For example, a representative study developed a dual‐branch network with a transformer to achieve precise segmentation of COVID‐19 infections in CT volumes [24].

The analysis of structured data (e.g., demographic, clinical, and laboratory), addressed in 178 studies, was applied across a wide spectrum of clinical tasks. A primary application was direct diagnostic and prognostic modeling, where specific supervised learning algorithms were employed to predict key clinical outcomes. Tabatabaie et al. [25] utilized ML on admission laboratory data to predict the mortality of COVID‐19 patients. A closely related objective was the identification of key risk factors; studies like that leveraged ML models to determine which clinical and laboratory variables were most important for mortality prediction [26]. This was exemplified by using clustering algorithms to discover novel patient subgroups from clinical data, identifying distinct COVID‐19 severity clusters based on laboratory parameters [27]. The power of combining these structured data streams with imaging was highlighted in multimodal systems, such as the one by Mei et al. [28], which integrated both data types to achieve diagnostic performance superior to human experts.

In addition to applications on established clinical data, our review identified distinct clusters of research that explored the use of novel data modalities for diagnostics. A notable group of studies (*n* = 11) utilized generative adversarial networks (GANs) to address the challenge of limited and imbalanced datasets. These models were primarily employed to synthesize or augment medical images for robust model training, as demonstrated by Goel et al. [29] in their work on improving COVID‐19 detection through GAN‐based data augmentation. Another cluster of research (*n* = 7) focused on data privacy and governance through the application of federated learning (FL). This approach enabled the collaborative training of models across multiple institutions without the need to centralize or share raw patient data. Finally, we identified a significant body of literature on applying ML to novel data modalities. These included studies utilizing spectroscopic methods (*n* = 6) for noninvasive malaria diagnosis [30] and audio‐based diagnostics (*n* = 13) that leveraged cough recordings for COVID‐19 screening, exemplified by the CR19 framework [31].

### 3.3. Zoonotic Epidemiology and Risk Assessment in Human Populations

This domain, comprising 315 publications, examines ML applications in epidemiological surveillance and forecasting, utilizing 16 distinct models across eight data types (Figure 4, epidemiology panel). Most studies concentrated on forecasting epidemiological indicators including incidence and mortality (*n* = 250), while a significant portion addressed spatiotemporal analysis or risk assessment (*n* = 75). Reflecting the broader research landscape, studies were predominantly focused on COVID‐19 (*n* = 264) and malaria (*n* = 14). The studies employed diverse algorithms (Figure 4 and Figure S2A), with epidemiological time series constituting the primary data source (*n* = 304), frequently augmented by socioeconomic data including mobility patterns (*n* = 87) and environmental data (*n* = 52). Artificial neural networks (ANNs, *n* = 101) and recurrent neural networks (RNNs, *n* = 97) demonstrated particular utility for time‐series epidemiological data analysis (Figure 4 and Figure S2B).

Methodologically, these studies fall into two primary paradigms: direct data‐driven modeling (*n* = 196) and hybrid modeling that fuses ML with mechanistic principles (*n* = 119). In the direct data‐driven approach, models learn predictive patterns directly from the data. The predominant application was forecasting epidemic trajectories, for which RNN and their variants emerged as the most common choice for time‐series analysis (Figure 4, epidemiology panel). For instance, Shahin and Almotairi [32] employed a bidirectional LSTM (BiLSTM) encoder‐decoder architecture to forecast daily confirmed cases, recoveries, and deaths during the COVID‐19 pandemic in Saudi Arabia. Spatiotemporal risk assessment constituted another major application, where supervised models like RF and various neural networks linked diverse predictors to disease risk. A compelling example is the work that developed a dynamic network with exogenous input (NARX) neural network to generate weekly, county‐level Zika risk predictions for the USA by integrating epidemiological data with air travel volumes and local mosquito presence [33]. Unsupervised learning was also utilized for spatial analysis (*n* = 16), as illustrated by Dieng et al. [34], who applied functional clustering to village‐level malaria incidence curves in Senegal, identifying three distinct seasonal patterns and thereby providing an evidence base for tailoring control interventions. Moreover, graph neural network (GNN, *n* = 12) has emerged as a powerful tool for analyzing transmission on network‐structured data. Their applications include learning local contagion rules from mobility data and identifying potential asymptomatic spreaders from genetic sequence networks [35, 36].

The second paradigm, hybrid modeling, was a key trend in 119 studies. This approach combines the epidemiological principles and interpretability of mechanistic models (e.g., SIR/SEIR) with the flexibility and predictive ability of ML. The most common integration pattern was AI‐augmented parameter inference, wherein ML algorithms dynamically estimate the time‐varying parameters of a mechanistic model. A representative study by Zheng et al. [37] used natural language processing (NLP) to extract features from news articles, which then informed an LSTM network that predicted the dynamic infection rate for an SI model of COVID‐19 in China. A second major strategy involves physics‐informed neural networks (PINNs, *n* = 22) and epidemiology‐aware AI models (*n* = 9), which embed the mechanistic model’s governing differential equations directly into the neural network’s loss function. Angeli et al. [38] utilized this approach to learn the parameters of a SAIVR model, enabling counterfactual simulations to assess the impact of different vaccination rollout speeds. The fusion concept also extends to more modular integrations, such as incorporating mechanistic models as features within ensemble frameworks (*n* = 7) or as surrogate models to accelerate calibration (*n* = 16). Finally, a notable trend spanning both paradigms is the use of real‐time digital data streams (*n* = 12). This data served not only as a leading indicator for forecasting—for example, using Google search trends to predict COVID‐19 incidence [39]—but also for more nuanced surveillance applications, such as applying NLP to Reddit posts to characterize the longitudinal evolution of self‐reported symptoms [40]. The integration of such novel data sources represents a significant advancement toward more responsive and fine‐grained public health surveillance systems.

### 3.4. Strategies for Intervention and Optimization in Human Populations

This domain encompasses 92 publications focused on developing and optimizing public health intervention strategies, utilizing 16 distinct models across seven data types (Figure 4, intervention panel). The research is primarily divided into two areas: broad public health policies and behavioral interventions (*n* = 71), including nonpharmaceutical interventions (NPIs), and optimal allocation of critical resources (*n* = 21), such as vaccines and ventilators. These two areas collectively form a comprehensive approach to population health management. Consistent with the review’s overall scope, studies predominantly centered on the COVID‐19 pandemic (*n* = 85). RL (*n* = 37), ANN (*n* = 33), RF (*n* = 16), and Boosting (*n* = 14) were the leading methodologies (Figure S2A), collectively accounting for 88% (81/92) of publications in this domain. These models primarily utilized epidemiological (*n* = 83) and socioeconomic data (*n* = 58) to inform their analyses, often integrating both data types (Figure 4 and Figure S2B).

The methodologies can be broadly categorized into two paradigms: evaluative frameworks and prescriptive frameworks. The evaluative approach employs ML methods, such as supervised learning and unsupervised learning, to assess situations for decision support. This approach generally has two aims: to assess intervention effectiveness or to identify priority populations. For instance, in evaluating NPIs, Tao et al. [41] used a RF model to analyze data from 147 countries, identifying body mass index, duration of facial covering mandates, and restrictions on small gatherings as the three most critical determinants of the second‐wave COVID‐19 effective reproduction number (*R*_*e*_). Unsupervised learning is also applied, for example, to cluster geographical regions with similar epidemiological profiles, thereby identifying high‐risk populations that require tailored control strategies [42]. Another key application of the evaluative approach involves resource prioritization. Manupati et al. [43] developed a multiphase vaccine distribution model wherein a decision tree classified Indian cities into different priority levels based on active cases and mortality rates, thereby guiding initial vaccine allocation. This approach has also been used to analyze the direct effects of vaccination campaigns. For example, Magazzino et al. [44] employed an ANN to analyze data from 192 countries, identifying the critical “cut effect” point at which vaccination campaigns began to reduce COVID‐19 fatality rates significantly.

Equally prominent is the prescriptive paradigm, which uses RL to find optimal, dynamic control strategies, often addressing complex trade‐offs between public health outcomes and economic costs. A common feature of these studies is the training of RL agents within complex simulations, frequently agent‐based models (*n* = 8). This approach is exemplified in the optimization of NPI policies, where Guo et al. [45] developed the PaCAR framework. Their system used a deep Q‐network (DQN) to learn strategies that balance infection control with minimal societal restrictions by selecting from four discrete NPI levels within a large‐scale ABM environment. Notably, many studies integrate these paradigms to create more robust decision‐support systems. For example, to optimize ventilator allocation, Bednarski et al. [46] developed a collaborative multiagent RL system where an LSTM (an evaluative model) first forecasted state‐level demand, and a Q‐learning agent (a prescriptive model) subsequently determined optimal interstate redistributions to minimize national shortages. These hybrid approaches highlight a shift towards proactive, data‐driven policymaking, enabling public health authorities to design and adapt interventions with enhanced foresight and precision.

### 3.5. Pathogen Detection in Animal Reservoirs and Vector Monitoring

**Figure 5:**
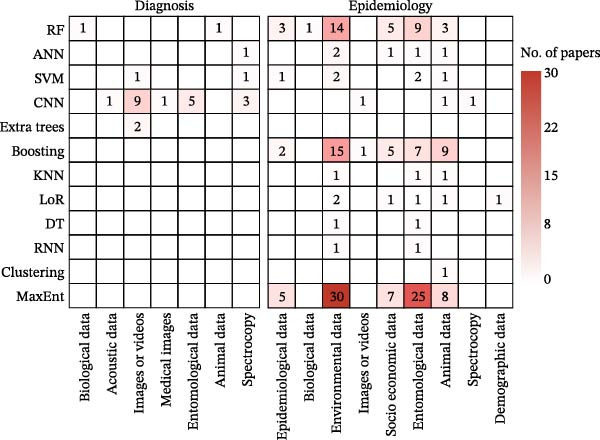
Co‐occurrence of ML models (rows) and data types (columns) applied to the management of animal reservoirs and vectors across functional domains (diagnosis and epidemiology). Cell values indicate the number of model‐data coapplications; a single publication may contribute to multiple cells if it utilized multiple models or data types. Color intensity reflects the number of papers. Abbreviations: ANN, artificial neural network; CNN, convolutional neural network; DT, decision tree; GAN, generative adversarial network; KNN, K‐nearest neighbors; LoR, logistic regression; MaxEnt, maximum entropy; NB, Naive Bayes; RF, random forest; RNN, recurrent neural network; GNN, graph neural network; SVM, support vector machine.

This section analyzes 18 publications that applied ML to diagnostic tasks in animal and vector surveillance, employing five distinct models across seven data types (Figure 5, diagnosis panel). The research addressed two critical applications: pathogen detection and diagnosis in animals (*n* = 5) and vector breeding site identification and species classification (*n* = 13). The primary data types included images or videos, entomological data, and spectroscopy (Figure 5 and Figure S3B). CNN dominated the methodological approaches (*n* = 12, Figure S3A), primarily applied to entomological or environmental images and videos. Other models, such as support vector machine (SVM), RF, and Boosting, are also frequently utilized.

A key application, investigated in five studies, is the early detection of pathogens in animal hosts to prevent zoonotic transmission. Among these approaches, wearable device technologies showed promising results. For example, Davis et al. [47] used RF models on physiological telemetry data from wearable devices on nonhuman primates, achieving AUC scores of 0.93 and 0.99 for detecting pathogen exposure. For reservoir species identification, four additional studies employed ML approaches to assist in pathogen detection in animal hosts and vectors. ML was used to enhance traditional laboratory assays. For example, traditional methods such as qPCR and LAMP assays are frequently used to detect Wolbachia in *Aedes aegypti* mosquitoes. However, these methods are susceptible to false negatives and may underestimate Wolbachia prevalence. Rakotonirina et al. [48] combined mass spectrometry with CNN for monitoring Wolbachia‐infected *Ae. aegypti* mosquitoes in field conditions. Results demonstrated that the CNN could recognize spectral patterns associated with Wolbachia infection, achieving high true positive rate (93%) and accuracy (97%).

Timely detection of vectors or their breeding sites is crucial in controlling infections. In this context, using entomological or environmental images to classify mosquitoes and their breeding sites has shown promise in improving global vector control programs and reducing vector‐borne infections. Five studies applied ML models with entomological images, sounds, or spectroscopy data to identify mosquito species, ages, sex, or breeding sites. For instance, Bravo et al. [49] compared deep neural networks (i.e., CNN YOLOv3) and traditional ML (i.e., BoVW + SVM) for identifying potential breeding sites in aerial drone images. CNN YOLOv3 demonstrated a mean average precision of 96.5%, outperforming BoVW + SVM in detection rates. Pataki et al. [50] examined ResNet50 models for identifying mosquitoes based on uploaded mosquito images from the Mosquito Alert platform. By training a ResNet50 model with various image augmentation techniques, including negative samples of nonmosquito insects, the authors achieved AUC scores of 0.96, providing a helpful preselector for expert validation. Beyond identification tasks, ML has also been translated into operational outbreak prediction tools. Modu et al. [51] deployed an intelligent malaria outbreak early warning system as a publicly available mobile application that predicts outbreak risk several days in advance using climatic data and an optimized SVM model (99% accuracy), representing one of the first field‐ready ML‐driven early warning tools for healthcare providers.

### 3.6. Spatial Patterns and Determinants of Zoonotic Diseases in Animals and Disease Vectors

This section analyzes 56 epidemiological modeling studies focused on animal reservoirs and vectors, employing 11 distinct models across nine data types (Figure 5, epidemiology panel). Traditional ML algorithms dominated the methodological landscape, with maximum entropy (MaxEnt) (*n* = 30) and Boosting (*n* = 15) being the most frequently employed (Figure S3A). Environmental data (*n* = 53) and entomological data (*n* = 39) constituted the primary inputs (Figure S3B). The applications primarily included three categories: modeling animal reservoir and vector population (*n* = 38), predicting pathogen infection and outbreak (*n* = 17), and identifying risk factors (*n* = 16).

The largest category of applications was vector distribution and population modeling, wherein ML models provide computationally efficient alternatives to traditional stochastic approaches. Kinney et al. [52] developed ANN‐based models to predict mosquito abundance from local weather time series, achieving significant computational efficiency gains. Bhattachan et al. [53] applied spatiotemporal RF models to assess water use restriction impacts on mosquito populations, revealing effective abundance control. For spatial distribution, Moua et al. [54] used MaxEnt with environmental and anthropogenic data to predict the presence of *Anopheles darlingi*, achieving a high cross‐validated AUC of 0.93. Comparative analyses were also common. Liu et al. [55] simulated *Anopheles dirus* distribution under future climate scenarios using RF, boosted regression trees (BRTs), and MaxEnt, finding negligible performance differences among the models. A second research focus addressed risk factor identification and pathogen outbreak prediction. Risk factor analyses, which often used RF (*n* = 4), consistently identified climate variables (e.g., temperature and precipitation) as critical predictors of vector populations and pathogen transmission. For outbreak prediction, studies have integrated diverse data to identify high‐risk regions and transmission determinants. Walsh applied MaxEnt to model RVF risk across Africa, integrating biotic and abiotic landscape features. The model identified intermittent wetlands and host species density as key predictors and demonstrated robust performance against independent test data [56].

### 3.7. Predicting Spillover Risk at the Animal‐to‐Human Interface

**Figure 6:**
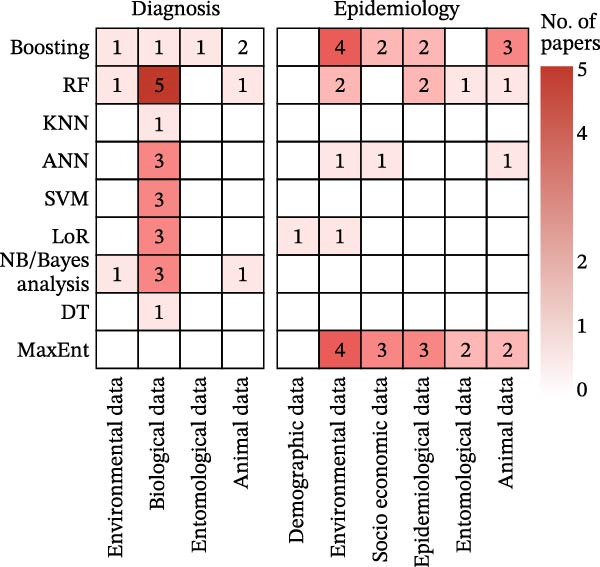
Co‐occurrence of ML models (rows) and data types (columns) applied to the management of the animal–human interface (spillover) across functional domains (diagnosis and epidemiology). Cell values indicate the number of model‐data coapplications; a single publication may contribute to multiple cells if it utilized multiple models or data types. Color intensity reflects the number of papers. Abbreviations: ANN, artificial neural network; DT, decision tree; KNN, K‐nearest neighbors; LoR, logistic regression; MaxEnt, maximum entropy; NB, Naive Bayes; RF, random forest; SVM, support vector machine.

This domain comprises 19 publications focused on pathogen spillover from animals to humans, using nine distinct models applied to seven data types (Figure 6). These studies are divided into diagnostic modeling for analyzing pathogen spillover source and infectivity (*n* = 11) and epidemiological modeling for predicting spillover risk and environmental suitability (*n* = 8). RF models predominated in diagnostic tasks (Figure S4A, *n* = 4), and biological data were the primary input in these articles (Figure S4B, *n* = 8). For epidemiological tasks, MaxEnt emerged as the most common model (Figure S4A, *n* = 4), and these studies mainly employed environmental data (Figure S4B, *n* = 11). Further stratification of the spillover studies suggested marked imbalances across transmission modes, geographic contexts, and functional domains. Vector‐borne diseases constituted the largest share (*n* = 9), followed by direct contact (*n* = 5) and airborne/droplet diseases (*n* = 5). Geographically, all spillover studies originated from HICs (*n* = 14, 73.7%) or UMICs (*n* = 5, 26.3%), with no study conducted in an LMIC, in contrast with the human population stage where LMICs contributed 21.4% of studies. By functional domain, spillover studies covered only diagnosis (*n* = 11) and epidemiology (*n* = 8), with no intervention model targeting spillover prevention.

Diagnostic applications focus on identifying high‐risk animal hosts and pathogens with high zoonotic potential. The RF model–biological data combination was most common (*n* = 5, Figure 6). A primary application involves identifying potential animal reservoirs and vectors. One study used a generalized boosted regression model to prioritize Nipah virus surveillance by identifying new bat species likely exposed to the virus [57]. Similarly, Evans et al. [58] developed ecological network models linking vector‐virus traits to identify unknown Zika vectors, predicting 35 potential vector species. Moreover, ML models for host susceptibility mapping achieved 80%–97% accuracy across diverse pathogen systems, including coronavirus‐bat, West Nile virus (WNV)‐bird, and Ebolavirus‐mammal associations [59–61]. Another key diagnostic function is to characterize the zoonotic potential and origin of pathogens, often through genomic analysis. In one study, an ANN analyzed spike protein sequences, demonstrating high accuracy in predicting human receptor binding potential and successfully identifying previously unknown human‐binding coronaviruses [62]. Notably, this model accurately predicted outcomes for SARS‐CoV‐2 even when it was excluded from the training data [63]. For the source attribution of zoonotic infections, ML models analyzing viral gene sequences have effectively determined the host origins. In one study, a model differentiated infections caused by swine‐lineage A(H1N1) pdm09 virus from those caused by seasonal human strains with 85.7% to 100% accuracy [64]. Other studies have demonstrated the capability of ML to forecast pathogen infectivity, notable achievements include a novel CWBLR algorithm achieving 100% accuracy (AUC 0.99) for H7N9 human infectivity prediction [65], a CNN model achieving ~99% accuracy for H5Nx pathogenicity classification, and a wavelet‐based neural network that successfully discriminated molecular patterns associated with the interspecies transmission of avian influenza [62].

Epidemiological applications assess the spillover risk and identify contributing factors through diverse modeling approaches. The combination of MaxEnt and BRT with environmental data was most prevalent for these tasks (*n* = 4, Figure 4). For the Lassa virus, Basinski et al. [66] used a multilayer BRT framework to first predict the geographic range of the reservoir, *Mastomys natalensis*, and then correlate this risk with historical spillover patterns. Similarly, Shartova et al. [67] also applied MaxEnt to delineate transmission‐suitable environments using data on human infection sites, virus detection in hosts, NDVI, and road density. Several models have been applied to the Ebola virus assessment. Redding et al. [68] developed an environmental‐mechanistic model, which uses ML (BRTs) to predict host distribution and then integrates this information with ecological, epidemiological, and socioeconomic factors into a mechanistic model to predict spillover risk and subsequent epidemic potential. Mursel et al. [69] defined a spillover risk index using GBDT, XGBoost, and logistic regression (LoR) on sociodemographic survey data to quantify individual exposure risk. Shapiro et al. [70] employed GLM and MaxEnt to investigate spillover determinants, revealing associations with bat species richness and low anthropogenic disturbance. For WNV, Keyel et al. [71] used RF to link mosquito and human infection rates with seasonal climate and hydrology, while Sallam et al. [72] applied MaxEnt in New Orleans to model vector‐host contact ratios, demonstrating land use, climate, and socioeconomic influences on vector distribution.

## 4. Discussion

This review presents a comprehensive analytical framework examining ML applications across different epidemiological stages. Although nearly 90% of publications appeared between 2019 and 2023, this surge was mainly driven by the COVID‐19 pandemic, which accounted for 73% of all studies (Figure 1b). Critical gaps persist in understanding and intervening at the animal–human interface, with no intervention models addressing animal hosts or pathogen spillover. This pattern reflects a reactive rather than preventive approach to zoonotic disease management. These findings highlight the urgent need to balance research priorities and develop integrated modeling frameworks capable of anticipating zoonotic threats, rather than merely responding to outbreaks after they emerge in human populations. Our findings provide an interdisciplinary roadmap that integrates epidemiological, ecological, data science, and social science expertise, identifying critical research gaps and facilitating collaborative approaches necessary for comprehensive zoonotic disease management.

The majority of reviewed publications tend to emphasize the importance of increased investment in technology for diagnosing, treating, and vaccinating against emerging diseases, often overlooking the significance of using ML techniques to predict and mitigate spillover risks at various scales. This suggests that current disease management frameworks may be disproportionately reactive rather than preventive. In addition, our stratified analysis indicates that this gap varies systematically by the transmission mode and geographic context. Policymakers should therefore establish specific funding mechanisms tailored to the distinct data landscapes and transmission dynamics of each disease category and institutionalize One Health surveillance systems that bridge the human, animal, and environmental health sectors [73, 74]. Vector‐borne diseases constitute the largest share of spillover studies, while existing models rely mainly on species distribution approaches with environmental predictors, resulting in the critical link between vector dynamics and actual cross‐species transmission largely unaddressed. Transfer learning methods that leverage well‐studied diseases to forecast related pathogens in data‐scarce settings represent a promising but unexplored avenue [75]. For direct‐contact zoonoses, diseases such as monkeypox (31 human‐stage studies) and HIV (22 human‐stage studies) have received few attention at the spillover stage, despite their documented zoonotic origins. Spillover of these pathogens is shaped by human‐wildlife behavioral interactions, including bushmeat hunting, wildlife trade, and livestock management practices, that are difficult to capture with environmental data alone. Recent geospatial modeling has demonstrated that mammal species richness and deforestation are principal drivers of bushmeat activity distribution [76], yet such sociobehavioral predictors have not been incorporated into ML‐based spillover risk frameworks. Airborne/droplet pathogens, particularly avian influenza, ML models can now predict zoonotic potential from viral genomic sequences, but recent benchmarking has revealed blind spots in key lineages including H5 influenza A viruses [77], highlighting the need to integrate genomic features with ecological surveillance data.

Across all three categories, the absence of spillover research originating from LMICs is notable, given that these regions have the high risk of zoonotic emergence yet lack sufficient surveillance infrastructure [78]. This geographic concentration also raises concerns about the global applicability of the existing spillover models. A previous study suggested that, after correcting for reporting bias, zoonotic emerging infectious disease risk is highest in forested tropical regions of sub‐Saharan Africa, Latin America, and Southeast Asia undergoing land‐use change, yet global scientific and surveillance resources are disproportionately concentrated in higher‐income countries, creating a significant mismatch between research investment and emergence risk [78]. Our findings also reflect this mismatch: All 19 spillover studies originated from HICs or UMICs, while LMICs, which contributed 21.4% of human‐stage studies, had no representation at the spillover stage. Within a One Health framework [73], this mismatch weakens early warning ability precisely where integrated surveillance is most needed. Addressing this disparity needs lightweight, offline‐capable ML tools suited to low‐infrastructure environments, complemented by community‐based surveillance platforms [79]. Recent advances demonstrated that combining statistical hotspot models with participatory expert workshops in five LMICs (Kenya, Peru, Senegal, Thailand, and Vietnam) can reduce surveillance blind spots that neither approach addresses alone. Future efforts should prioritize international partnerships that support local data generation and participatory model development in high‐burden LMICs [80].

Intervention models targeting animal reservoirs or pathogen spillover were notably absent from the reviewed literature; a parallel systematic review of policies to prevent zoonotic spillover similarly found that evaluations of interventions targeting the determinants of spillover remain extremely scarce, in part because the complex, multihost systems in which spillover occurs make both intervention design and evaluation particularly challenging [81] and because spatially and temporally explicit data linking reservoir ecology, host behavior, and viral dynamics at the point of spillover are rarely available [82]. Even within human populations, most intervention studies remain theoretical, with limited demonstrations of practical applicability. Several modeling assumptions widen the gap between theoretical optimization and real‐world use. Disease transmissibility, though modeled as dynamic, is frequently calibrated using lagged and potentially incomplete surveillance data. Human contact patterns are often assumed to be static, and the impact of interventions is typically modeled using linear terms, failing to fully capture the complex interplay between public responses and pathogen evolution. More broadly, most studies formulate the optimization problem using oversimplified assumptions about disease transmission dynamics, decision‐making processes, and the costs and impacts of intervention strategies [10], and translating the resulting policies into simple rules that can be read and interpreted by human decision‐makers remains a challenge [83]. Addressing these limitations across the full transmission pathway, from upstream spillover determinants to human population control, remains an important priority for future research [84, 85].

Performance metrics such as AUC and accuracy reported across the reviewed studies should be read in light of differences in the validation approach and sample size, which affect how reliably these results reflect model behavior on new data. Models evaluated through internal cross‐validation are tested on data drawn from the same distribution as the training set, which tends to produce more favorable estimates than testing on independent data. The gap between the two can be significant; for example, a model predicting H7N9 human infectivity reported near‐perfect performance on its training cohort, but external validation on strains collected in a later period revealed 100% accuracy for human‐origin strains and only 50% for avian‐origin strains [65]. The validation strategy also affects how stable an estimate is within a single study: A mosquito identification model reported AUC = 0.96 on a held‐out test set, yet AUC ranged from 0.90 to 0.93 across yearly cross‐validation folds, reflecting sensitivity to variation in image quality and class composition over time [50]. Beyond validation design, sample size shapes how much weight a reported figure can carry. An AUC of 0.93 derived from a small cohort of laboratory animals under tightly controlled experimental conditions reflects a different level of evidence than one obtained from a large, field‐collected dataset, even when the numbers appear comparable [47]. These differences do not diminish the value of individual studies, but they do suggest that the metrics reported across this review should not be treated as directly comparable indicators of model performance.

Despite most reviewed studies remain at the model development and validation stage, a few examples demonstrate successful real‐world deployment. Bastani et al. [86] reported Eva, a RL system deployed across all 40 Greek border entry points from August to November 2020 to optimize COVID‐19 testing allocation for asymptomatic travelers. Developed in collaboration with the Greek COVID‐19 Executive Committee, Eva identified 1.85 times as many infected travelers as random surveillance testing during peak season and directly informed national border policy decisions, illustrating how ML‐driven surveillance tools can be operationalized at scale when combined with institutional collaboration and interpretable algorithm design. However, we note that integrating nontraditional surveillance data sources, including social media content, medical reports, and satellite imagery, remains challenging.

The predominance of COVID‐19–related studies in reviewed publications also represents a significant limitation. SARS‐CoV‐2’s unique characteristics, its high transmission efficiency, and the unprecedented global public health response it elicited have driven the development of modeling approaches that may prove less applicable to pathogens with different ecological niches, reservoirs, or transmission dynamics. The extensive resource mobilization during the COVID‐19 pandemic facilitated data collection and model development at scales rarely achieved for other zoonoses, potentially creating unrealistic expectations regarding data availability for future ML applications in less‐studied diseases. In particular, the prominence of DL in this review reflects the data conditions specific to COVID‐19, including large‐scale clinical imaging, electronic health records, and real‐time epidemiological surveillance streams, rather than a generalizable advantage across the zoonotic disease spectrum. For diseases where surveillance data are ecological, serological, or limited in volume, the choice of analytic method should be matched to the available data structure rather than inherited from a pandemic context [87]. This constraint is further amplified at the animal reservoir stage, where geographical and taxonomic biases in host‐pathogen association records substantially undermine model reliability [15]. For example, recent advances in remote sensing technology, particularly when coupled with AI‐based image analysis, have enabled rich information extraction from satellite data. Nevertheless, monitoring small animal populations that serve as disease vectors presents challenges. Rodents and birds, whose population distributions influence vector‐borne disease transmission, exhibit high seasonal fluctuations and mobility that complicate accurate data collection. Under such conditions, network‐based approaches are often confounded by sampling bias and restricted to in‐sample prediction, whereas trait‐based ensemble methods have demonstrated greater out‐of‐sample predictive capacity [88]. Most methodological frameworks depend on adequate, high‐quality data; the concentration of research on specific diseases such as COVID‐19 and malaria underscores the need for policy frameworks that encourage research on neglected and emerging zoonoses, particularly in low‐ and middle‐income countries with limited surveillance capacity. For instance, GNN‐based methods require individual‐level contact network data, which is typically difficult to obtain.

Beyond data scarcity and quality, including the challenges of animal reservoir surveillance discussed above, effective data integration faces additional barriers rooted in privacy constraints and format heterogeneity. Privacy legislation such as GDPR and HIPAA prevents centralized pooling of health data across institutions and borders [89], while the lack of standardized metadata across repositories creates pervasive interoperability gaps that impede data discoverability and reuse [90]. Addressing these challenges requires targeted strategies that tackle three interrelated barriers: data privacy constraints, format heterogeneity, and low‐quality animal reservoir data. Privacy regulations (e.g., GDPR and HIPAA) restrict centralized pooling of clinical records across institutions and borders. FL offers a promising solution by enabling collaborative model training on distributed datasets without transferring raw data. A recent study found that FL achieved prediction accuracy comparable to centralized approaches across multiple infectious disease applications, though most studies relied on manually partitioned rather than real‐world federated data [89]. In addition, the human health, animal health, and environmental surveillance sectors use heterogeneous data formats and terminologies that impede interoperability. Standardized metadata schemas based on the FAIR principles have been developed for infectious disease datasets [90], and a zoonotic diseases minimum dataset (ZD‐MDS) mapped to SNOMED‐CT clinical terminology and structured via the HL7‐CDA standard provides a disease‐specific solution [91]. Animal reservoir data also suffer from geographical and taxonomic sampling biases, with unstudied species frequently misclassified as nonhosts. Recent advances have introduced modeling frameworks that mitigate such uncertainty through “pseudo‐negative” species identification and bias‐aware prediction pipelines [88], while ensemble approaches combining trait‐based and network models, with systematic validation against newly discovered hosts, have shown that iterative prediction and updating can improve the prioritization of viral discovery in zoonotic reservoirs [92].

The work present here categorized studies by algorithm type and functional domain but did not systematically assess the adoption of interpretability or causal inference methods, which is a limitation. These two dimensions are increasingly recognized as essential for translating ML into public health practice. A recent systematic review of AI‐driven infectious disease early warning systems found that the opacity of complex models remains one of the most significant barriers to their operational adoption, as public health professionals need to understand and validate model outputs before acting on them [93]. Explainable AI techniques such as SHAP and LIME can help address this barrier by quantifying feature‐level contributions to predictions. At the same time, causal inference is attracting growing attention in epidemiological ML research. New doubly‐robust estimators, including targeted maximum likelihood estimation (TMLE), augmented inverse probability weighting, and double/debiased ML, now allow researchers to harness the predictive flexibility of ML while producing unbiased causal effect estimates from observational data [94]. Future reviews of ML in zoonotic disease research should consider incorporating interpretability and causal structure as explicit analytical dimensions.

Our literature search and screening were completed in mid‐2023; given the rapid pace of development in the field, some recent advances may not be fully reflected in this review. In particular, large language models (LLMs) and multimodal approaches have emerged as a distinct methodological frontier since then. These methods have demonstrated the ability to integrate heterogeneous inputs such as epidemiological time series, surveillance reports, and policy text for real‐time disease forecasting [95], and their broader implications for epidemic modeling have been systematically discussed [96]. These advances are largely concentrated in human‐stage applications where data are abundant; their relevance to spillover‐stage modeling, where documented events remain rare and training data limited [97], is currently less clear. Beyond individual disease modeling, AI is increasingly being applied to the meta‐analysis of scientific literature to complement traditional review methods. ML has been used to map knowledge structures across research fields, identify neglected topics, reveal geographic or thematic biases, and support attribution inferences by integrating evidence from different domains [77]. Applying these approaches in zoonotic disease research could enable more timely and comprehensive synthesis in the future. Addressing the critical gaps at the animal–human interface will ultimately require integrated modeling frameworks that combine ecological, behavioral, and epidemiological data sources, alongside international efforts to support local data generation and model development in high‐burden regions [10].

## 5. Conclusions

This scoping review reveals a critical imbalance in ML applications across the zoonotic disease transmission pathway. Research is concentrated on postspillover response in human populations, while surveillance at animal reservoirs and the animal–human interface remains underexplored. The COVID‐19 pandemic triggered a reactive surge that accounts for most of the reviewed studies, and the heavy reliance on DL shaped by the large‐scale clinical and surveillance data available during this period may not generalize to other zoonoses. Although integrated models combining ML with mechanistic epidemiological frameworks are a promising methodological trend, challenges related to model interpretability and causal inference continue to limit translation into public health practice. Moreover, our stratified analysis shows that spillover dynamics and data availability differ significantly across transmission modes, requiring that future modeling strategies account for the distinct ecological, behavioral, and data characteristics of each disease category. The absence of spillover research from low‐ and LMICs, despite their high emerging risk, highlights the need for lightweight, offline‐capable ML tools and international partnerships that support local data generation. Future research should develop integrated ML frameworks that span the entire transmission pathway, strengthen cross‐sectoral data integration between the human and animal health sectors, and support participatory model development in high‐burden regions to enable the proactive spillover prevention that a One Health approach demands.

## Author Contributions

Sen Li and Yinsheng Zhang designed the study. Yinsheng Zhang completed database searching. Yinsheng Zhang, Yifan Sun, Jinchen Wang, Ruying Fang, Luqi Wang, Xiaolong Wu, Yiyang Guo, and Xin Yang performed the study. Yinsheng Zhang, Yifan Sun, Jinchen Wang, and Ruying Fang analyzed the data. All authors contributed to conflict resolution during screening. Yinsheng Zhang and Yifan Sun synthesized the findings and wrote the original manuscript. Sen Li critically revised the manuscript and provided senior advice.

## Funding

This study was supported by the National Natural Science Foundation of China (Grants 42477465, 42311530697).

## Disclosure

All authors contributed to reviewing and editing of the final manuscript and approved the final version of the manuscript.

## Ethics Statement

The authors confirm that they have adhered to the ethical policies of the journal, as noted on its author guidelines page. As this is a scoping review article with no original research data, ethical approval was not required.

## Conflicts of Interest

The authors declare no conflicts of interest.

## Supporting Information

Additional supporting information can be found online in the Supporting Information section.

## Supporting information


**Supporting Information** Text S1: Search strategies for databases of publications. Figure S1: Flow diagram of the selection procedure. Figure S2: Data types and machine learning models applied to management in human populations. Figure S3: Data types and machine learning models applied to management in animal reservoirs and vectors. (A) Chord diagram showing the distribution of ML models across the functional domains of diagnosis, epidemiology, and intervention. The numbers indicate the total count of model applications within each application domain. (B) Chord diagram showing the distribution of data types across the same functional domains. The numbers indicate the total count of applications of the data types within each domain. Figure S4: Data types and machine learning models applied to management in animal–human interface. (A) Chord diagram showing the distribution of ML models across the functional domains of diagnosis, epidemiology, and intervention. The numbers indicate the total count of model applications within each application domain. (B) Chord diagram showing the distribution of data types across the same functional domains. The numbers indicate the total count of applications of the data types within each domain. Table S1: Epidemiological characteristics of the zoonotic diseases analyzed in this scoping review. Table S2: Summary of algorithm–data type coapplications organized by epidemiological stage and functional domain. Table S3: Representative borderline cases with prediction targets, assigned stages, and classification rationale.

## Data Availability

The data extracted from the scoping review are available from the corresponding author upon request.

## References

[1] Oeschger T. M. , McCloskey D. S. , and Buchmann R. M. , et al.Early Warning Diagnostics for Emerging Infectious Diseases in Developing Into Late-Stage Pandemics, Accounts of Chemical Research. (2021) 54, no. 19, 3656–3666, 10.1021/acs.accounts.1c00383. 34524795

[2] Jones K. E. , Patel N. G. , and Levy M. A. , et al.Global Trends in Emerging Infectious Diseases, Nature. (2008) 451, no. 7181, 990–993, 10.1038/nature06536, 2-s2.0-39749135587. 18288193 PMC5960580

[3] Doran Á. , Colvin C. L. , and McLaughlin E. , What can We Learn From Historical Pandemics? A Systematic Review of the Literature, Social Science & Medicine. (2024) 342, 10.1016/j.socscimed.2023.116534, 116534. 38184966

[4] Karesh W. B. , Dobson A. , and Lloyd-Smith J. O. , et al.Ecology of Zoonoses: Natural and Unnatural Histories, The Lancet. (2012) 380, no. 9857, 1936–1945, 10.1016/S0140-6736(12)61678-X, 2-s2.0-84870261809. PMC7138068 23200502

[5] Carvalho R. L. , Anjos D. , and Harmange C. , et al.Unpacking the Risks of Zoonotic and Vector-Borne Pathogen Transmission to Humans in the Context of Environmental Change, One Earth. (2025) 8, no. 8, 10.1016/j.oneear.2025.101348, 101348.

[6] Bernstein A. S. , Ando A. W. , and Loch-Temzelides T. , et al.The Costs and Benefits of Primary Prevention of Zoonotic Pandemics, Science Advances. (2022) 8, no. 5, 10.1126/sciadv.abl4183, eabl4183. 35119921 PMC8816336

[7] Rulli M. C. , D’Odorico P. , Galli N. , and Hayman D. T. S. , Land-Use Change and the Livestock Revolution Increase the Risk of Zoonotic Coronavirus Transmission From Rhinolophid Bats, Nature Food. (2021) 2, no. 6, 409–416, 10.1038/s43016-021-00285-x. 37118224

[8] Lloyd-Smith J. O. , George D. , and Pepin K. M. , et al.Epidemic Dynamics at the Human–Animal Interface, Science. (2009) 326, no. 5958, 1362–1367, 10.1126/science.1177345, 2-s2.0-71549151317. 19965751 PMC3891603

[9] Li S. , Gilbert L. , Vanwambeke S. O. , Yu J. , Purse B. V. , and Harrison P. A. , Lyme Disease Risks in Europe Under Multiple Uncertain Drivers of Change, Environmental Health Perspectives. (2019) 127, no. 6, 10.1289/EHP4615, 2-s2.0-85068542514, 67010. 31232609 PMC6792373

[10] Ye Y. , Pandey A. , and Bawden C. , et al.Integrating Artificial Intelligence With Mechanistic Epidemiological Modeling: A Scoping Review of Opportunities and Challenges, Nature Communications. (2025) 16, no. 1, 10.1038/s41467-024-55461-x, 581. PMC11724045 39794317

[11] Farooq Z. , Rocklov J. , and Wallin J. , et al.Artificial Intelligence to Predict West Nile Virus Outbreaks With Eco-Climatic Drivers, The Lancet Regional Health—Europe. (2022) 17, 10.1016/j.lanepe.2022.100370, 100370. 35373173 PMC8971633

[12] Guo Y. , Zhang Y. , and Lyu T. , et al.The Application of Artificial Intelligence and Data Integration in COVID-19 Studies: A Scoping Review, Journal of the American Medical Informatics Association. (2021) 28, no. 9, 2050–2067, 10.1093/jamia/ocab098. 34151987 PMC8344463

[13] Badillo S. , Banfai B. , and Birzele F. , et al.An Introduction to Machine Learning, Clinical Pharmacology & Therapeutics. (2020) 107, no. 4, 871–885, 10.1002/cpt.1796. 32128792 PMC7189875

[14] Han B. A. , O’Regan S. M. , Schmidt J. P. , Drake J. M. , and Bates A. , Integrating Data Mining and Transmission Theory in the Ecology of Infectious Diseases, Ecology Letters. (2020) 23, no. 8, 1178–1188, 10.1111/ele.13520. 32441459 PMC7384120

[15] Schuler J. , Hudson M. , Schwartz D. , and Samudrala R. , A Systematic Review of Computational Drug Discovery, Development, and Repurposing for Ebola Virus Disease Treatment, Molecules. (2017) 22, no. 10, 10.3390/molecules22101777, 2-s2.0-85032636921, 1777. 29053626 PMC6151658

[16] Albahri O. S. , Zaidan A. A. , and Albahri A. S. , et al.Systematic Review of Artificial Intelligence Techniques in the Detection and Classification of COVID-19 Medical Images in Terms of Evaluation and Benchmarking: Taxonomy Analysis, Challenges, Future Solutions and Methodological Aspects, Journal of Infection and Public Health. (2020) 13, no. 10, 1381–1396, 10.1016/j.jiph.2020.06.028. 32646771 PMC7328559

[17] Grignaffini F. , Simeoni P. , Alisi A. , and Frezza F. , Computer-Aided Diagnosis Systems for Automatic Malaria Parasite Detection and Classification: A Systematic Review, Electronics. (2024) 13, no. 16, 10.3390/electronics13163174, 3174.

[18] Rees E. M. , Minter A. , Edmunds W. J. , Lau C. L. , Kucharski A. J. , and Lowe R. , Transmission Modelling of Environmentally Persistent Zoonotic Diseases: A Systematic Review, The Lancet Planetary Health. (2021) 5, no. 7, e466–e478, 10.1016/S2542-5196(21)00137-6. 34245717

[19] Pike J. , Bogich T. , Elwood S. , Finnoff D. C. , and Daszak P. , Economic Optimization of a Global Strategy to Address the Pandemic Threat, Proceedings of the National Academy of Sciences. (2014) 111, no. 52, 18519–18523, 10.1073/pnas.1412661112, 2-s2.0-84924302008. PMC4284561 25512538

[20] Singh B. B. , Ward M. P. , and Dhand N. K. , Inherent Virus Characteristics and Host Range Drive the Zoonotic and Emerging Potential of Viruses, Transboundary and Emerging Diseases. (2022) 69, no. 4, e799–e813, 10.1111/tbed.14361. 34710290

[21] Piret J. and Boivin G. , Pandemics Throughout History, Frontiers in Microbiology. (2021) 11, 10.3389/fmicb.2020.631736, 631736. 33584597 PMC7874133

[22] Tricco A. C. , Lillie E. , and Zarin W. , et al.PRISMA Extension for Scoping Reviews (PRISMA-ScR): Checklist and Explanation, Annals of Internal Medicine. (2018) 169, no. 7, 467–473, 10.7326/M18-0850, 2-s2.0-85054287365. 30178033

[23] Apostolopoulos I. D. and Mpesiana T. A. , Covid-19: Automatic Detection From X-Ray Images Utilizing Transfer Learning With Convolutional Neural Networks, Physical and Engineering Sciences in Medicine. (2020) 43, no. 2, 635–640, 10.1007/s13246-020-00865-4. 32524445 PMC7118364

[24] Peng Y. , Zhang T. , and Guo Y. , Cov-TransNet: Dual Branch Fusion Network With Transformer for COVID-19 Infection Segmentation, Biomedical Signal Processing and Control. (2023) 80, 10.1016/j.bspc.2022.104366, 104366. 36415848 PMC9671472

[25] Tabatabaie M. , Sarrami A. H. , Didehdar M. , Tasorian B. , Shafaat O. , and Sotoudeh H. , Accuracy of Machine Learning Models to Predict Mortality in COVID-19 Infection Using the Clinical and Laboratory Data at the Time of Admission, Cureus. (2021) 13, no. 10, 10.7759/cureus.18768, e18768. 34804648 PMC8592290

[26] Wong K. C.-Y. , Xiang Y. , Yin L. , and So H.-C. , Uncovering Clinical Risk Factors and Predicting Severe COVID-19 Cases Using UK Biobank Data: Machine Learning Approach, JMIR Public Health and Surveillance. (2021) 7, no. 9, 10.2196/29544, e29544. 34591027 PMC8485986

[27] Benito-León J. , del Castillo M. D. , Estirado A. , Ghosh R. , Dubey S. , and Serrano J. I. , Using Unsupervised Machine Learning to Identify Age- and Sex-Independent Severity Subgroups Among Patients With COVID-19: Observational Longitudinal Study, Journal of Medical Internet Research. (2021) 23, no. 5, 10.2196/25988, e25988. 33872186 PMC8163491

[28] Mei X. , Lee H.-C. , and Diao K.-Y. , et al.Artificial Intelligence-Enabled Rapid Diagnosis of Patients With COVID-19, Nature Medicine. (2020) 26, no. 8, 1224–1228, 10.1038/s41591-020-0931-3. PMC7446729 32427924

[29] Goel T. , Murugan R. , Mirjalili S. , and Chakrabartty D. K. , Automatic Screening of COVID-19 Using an Optimized Generative Adversarial Network, Cognitive Computation. (2024) 16, no. 4, 1666–1681, 10.1007/s12559-020-09785-7. PMC7829098 33520007

[30] Adegoke J. A. , De Paoli A. , and Afara I. O. , et al.Ultraviolet/Visible and Near-Infrared Dual Spectroscopic Method for Detection and Quantification of Low-Level Malaria Parasitemia in Whole Blood, Analytical Chemistry. (2021) 93, no. 39, 13302–13310, 10.1021/acs.analchem.1c02948. 34558904

[31] Sharma A. and Mishra P. K. , Covid-MANet: Multi-Task Attention Network for Explainable Diagnosis and Severity Assessment of COVID-19 From CXR Images, Pattern Recognition. (2022) 131, 10.1016/j.patcog.2022.108826, 108826. 35698723 PMC9170279

[32] Shahin A. I. and Almotairi S. , A Deep Learning BiLSTM Encoding-Decoding Model for COVID-19 Pandemic Spread Forecasting, Fractal and Fractional. (2021) 5, no. 4, 10.3390/fractalfract5040175, 175.

[33] Akhtar M. , Kraemer M. U. G. , and Gardner L. M. , A Dynamic Neural Network Model for Predicting Risk of Zika in Real Time, BMC Medicine. (2019) 17, no. 1, 10.1186/s12916-019-1389-3, 2-s2.0-85071770137, 171. 31474220 PMC6717993

[34] Dieng S. , Michel P. , and Guindo A. , et al.Application of Functional Data Analysis to Identify Patterns of Malaria Incidence, to Guide Targeted Control Strategies, International Journal of Environmental Research and Public Health. (2020) 17, no. 11, 10.3390/ijerph17114168, 4168. 32545302 PMC7312547

[35] Murphy C. , Laurence E. , and Allard A. , Deep Learning of Contagion Dynamics on Complex Networks, Nature Communications. (2021) 12, no. 1, 10.1038/s41467-021-24732-2, 4720. PMC8342694 34354055

[36] Liu Z. , Ma Y. , Cheng Q. , and Liu Z. , Finding Asymptomatic Spreaders in a COVID-19 Transmission Network by Graph Attention Networks, Viruses. (2022) 14, no. 8, 10.3390/v14081659, 1659. 36016280 PMC9413350

[37] Zheng N. , Du S. , and Wang J. , et al.Predicting COVID-19 in China Using Hybrid AI Model, IEEE Transactions on Cybernetics. (2020) 50, no. 7, 2891–2904, 10.1109/TCYB.2020.2990162. 32396126

[38] Angeli M. , Neofotistos G. , Mattheakis M. , and Kaxiras E. , Modeling the Effect of the Vaccination Campaign on the COVID-19 Pandemic, Chaos, Solitons & Fractals. (2022) 154, 10.1016/j.chaos.2021.111621, 111621. 34815624 PMC8603113

[39] Ayyoubzadeh S. M. , Ayyoubzadeh S. M. , Zahedi H. , Ahmadi M. , and Kalhori S. R. N. , Predicting COVID-19 Incidence Through Analysis of Google Trends Data in Iran: Data Mining and Deep Learning Pilot Study, JMIR Public Health and Surveillance. (2020) 6, no. 2, 10.2196/18828, e18828. 32234709 PMC7159058

[40] Sarabadani S. , Baruah G. , Fossat Y. , and Jeon J. , Longitudinal Changes of COVID-19 Symptoms in Social Media: Observational Study, Journal of Medical Internet Research. (2022) 24, no. 2, 10.2196/33959, e33959. 35076400 PMC8852652

[41] Tao S. , Bragazzi N. L. , Wu J. , Mellado B. , and Kong J. D. , Harnessing Artificial Intelligence to Assess the Impact of Nonpharmaceutical Interventions on the Second Wave of the Coronavirus Disease 2019 Pandemic Across the World, Scientific Reports. (2022) 12, no. 1, 10.1038/s41598-021-04731-5, 944. 35042945 PMC8766477

[42] Bertozzi-Villa A. , Bever C. A. , and Gerardin J. , et al.An Archetypes Approach to Malaria Intervention Impact Mapping: A New Framework and Example Application, Malaria Journal. (2023) 22, no. 1, 10.1186/s12936-023-04535-0, 138. 37101269 PMC10131392

[43] Manupati V. K. , Schoenherr T. , Subramanian N. , Ramkumar M. , Soni B. , and Panigrahi S. , A Multi-Echelon Dynamic Cold Chain for Managing Vaccine Distribution, Transportation Research Part E: Logistics and Transportation Review. (2021) 156, 10.1016/j.tre.2021.102542, 102542. 34815731 PMC8602632

[44] Magazzino C. , Mele M. , and Coccia M. , A Machine Learning Algorithm to Analyse the Effects of Vaccination on COVID-19 Mortality, Epidemiology and Infection. (2022) 150, 10.1017/S0950268822001418, e168. 36093862 PMC9551183

[45] Guo X. , Chen P. , and Liang S. , et al.PaCAR: COVID-19 Pandemic Control Decision Making via Large-Scale Agent-Based Modeling and Deep Reinforcement Learning, Medical Decision Making. (2022) 42, no. 8, 1064–1077, 10.1177/0272989X221107902. 35775610

[46] Bednarski B. P. , Singh A. D. , and Jones W. M. , On Collaborative Reinforcement Learning to Optimize the Redistribution of Critical Medical Supplies Throughout the COVID-19 Pandemic, Journal of the American Medical Informatics Association. (2021) 28, no. 4, 874–878, 10.1093/jamia/ocaa324. 33295626 PMC7799039

[47] Davis S. , Milechin L. , and Patel T. , et al.Detecting Pathogen Exposure During the Non-Symptomatic Incubation Period Using Physiological Data: Proof of Concept in Non-Human Primates, Frontiers in Physiology. (2021) 12, 10.3389/fphys.2021.691074, 691074. 34552498 PMC8451540

[48] Rakotonirina A. , Caruzzo C. , and Ballan V. , et al.Wolbachia Detection in *Aedes aegypti* Using MALDI-TOF MS Coupled to Artificial Intelligence, Scientific Reports. (2021) 11, no. 1, 10.1038/s41598-021-00888-1, 21355. 34725401 PMC8560810

[49] Bravo D. T. , Lima G. A. , Luz W. A. , and Alves etal , Automatic Detection of Potential Mosquito Breeding Sites From Aerial Images Acquired by Unmanned Aerial Vehicles, Computers, Environment and Urban Systems. (2021) 90, 10.1016/j.compenvurbsys.2021.101692, 101692.

[50] Pataki B. A. , Garriga J. , Eritja R. , Palmer J. R. B. , Bartumeus F. , and Csabai I. , Deep Learning Identification for Citizen Science Surveillance of Tiger Mosquitoes, Scientific Reports. (2021) 11, no. 1, 10.1038/s41598-021-83657-4, 4718. 33633197 PMC7907246

[51] Modu B. , Polovina N. , Lan Y. , Konur S. , Asyhari A. T. , and Peng Y. , Towards a Predictive Analytics-Based Intelligent Malaria Outbreak Warning System, Applied Sciences. (2017) 7, no. 8, 10.3390/app7080836, 2-s2.0-85027699126, 836.

[52] Kinney A. C. , Current S. , Lega J. , and Stowell D. , Aedes-AI: Neural Network Models of Mosquito Abundance, PLoS Computational Biology. (2021) 17, no. 11, 10.1371/journal.pcbi.1009467, e1009467. 34797822 PMC8641871

[53] Bhattachan A. , Skaff N. K. , Irish A. M. , Vimal S. , Remais J. V. , and Lettenmaier D. P. , Outdoor Residential Water use Restrictions During Recent Drought Suppressed Disease Vector Abundance in Southern California, Environmental Science & Technology. (2021) 55, no. 1, 478–487, 10.1021/acs.est.0c05857. 33322894 PMC9426289

[54] Moua Y. , Roux E. , and Girod R. , et al.Distribution of the Habitat Suitability of the Main Malaria Vector in French Guiana Using Maximum Entropy Modeling, Journal of Medical Entomology. (2016) 54, no. 3, 606–621, 10.1093/jme/tjw199, 2-s2.0-85026360753. 28011731

[55] Liu X. , Song C. , Ren Z. , and Wang S. , Predicting the Geographical Distribution of Malaria-Associated *Anopheles dirus* in the South-East Asia and Western Pacific Regions Under Climate Change Scenarios, Frontiers in Environmental Science. (2022) 10, 10.3389/fenvs.2022.841966, 841966.

[56] Walsh M. G. , Willem de Smalen A. , Mor S. M. , and Althouse B. , Wetlands, Wild Bovidae Species Richness and Sheep Density Delineate Risk of Rift Valley Fever Outbreaks in the African Continent and Arabian Peninsula, PLoS Neglected Tropical Diseases. (2017) 11, no. 7, 10.1371/journal.pntd.0005756, 2-s2.0-85026721891, e0005756. 28742814 PMC5526521

[57] Plowright R. K. , Becker D. J. , and Crowley D. E. , et al.Prioritizing Surveillance of Nipah Virus in India, PLoS Neglected Tropical Diseases. (2019) 13, no. 6, 10.1371/journal.pntd.0007393, 2-s2.0-85068978028, e0007393. 31246966 PMC6597033

[58] Evans M. V. , Dallas T. A. , Han B. A. , Murdock C. C. , and Drake J. M. , Data-Driven Identification of Potential Zika Virus Vectors, eLife. (2017) 6, 10.7554/eLife.22053, 2-s2.0-85014882567, e22053. 28244371 PMC5342824

[59] Robles-Fernandez A. L. , Santiago-Alarcon D. , and Lira-Noriega A. , Wildlife Susceptibility to Infectious Diseases at Global Scales, Proceedings of the National Academy of Sciences. (2022) 119, no. 35, 10.1073/pnas.2122851119, e2122851119. PMC9436312 35994656

[60] Han B. A. , Majumdar S. , and Calmon F. P. , et al.Confronting Data Sparsity to Identify Potential Sources of Zika Virus Spillover Infection Among Primates, Epidemics. (2019) 27, 59–65, 10.1016/j.epidem.2019.01.005, 2-s2.0-85066240715. 30902616

[61] Schmidt J. P. , Maher S. , Drake J. M. , Huang T. , Farrell M. J. , and Han B. A. , Ecological Indicators of Mammal Exposure to Ebolavirus, Philosophical Transactions of the Royal Society B: Biological Sciences. (2019) 374, no. 1782, 10.1098/rstb.2018.0337, 2-s2.0-85071280692, 20180337. PMC6711296 31401967

[62] Qiang X. and Kou Z. , Prediction of Interspecies Transmission for Avian Influenza A Virus Based on a Back-Propagation Neural Network, Mathematical and Computer Modelling. (2010) 52, no. 11-12, 2060–2065, 10.1016/j.mcm.2010.06.008, 2-s2.0-77956341225.

[63] Gonzalez-Isunza G. , Jawaid M. Z. , Liu P. , Cox D. L. , Vazquez M. , and Arsuaga J. , Using Machine Learning to Detect Coronaviruses Potentially Infectious to Humans, Scientific Reports. (2023) 13, no. 1, 10.1038/s41598-023-35861-7, 9319. 37291260 PMC10248971

[64] Cook P. W. , Stark T. , and Jones J. , et al.Detection and Characterization of Swine Origin Influenza A(H1N1) Pandemic 2009 Viruses in Humans Following Zoonotic Transmission, Journal of Virology. (2020) 95, no. 2, e01066–01020, 10.1128/JVI.01066-20. 33115872 PMC7944445

[65] Sun Y. , Zhang K. , and Qi H. , et al.Computational Predicting the Human Infectivity of H7N9 Influenza Viruses Isolated From Avian Hosts, Transboundary and Emerging Diseases. (2021) 68, no. 2, 846–856, 10.1111/tbed.13750. 32706427 PMC8246913

[66] Basinski A. J. , Fichet-Calvet E. , and Sjodin A. R. , et al.Bridging the Gap: Using Reservoir Ecology and Human Serosurveys to Estimate Lassa Virus Spillover in West Africa, PLoS Computational Biology. (2021) 17, no. 3, 10.1371/journal.pcbi.1008811, e1008811. 33657095 PMC7959400

[67] Shartova N. , Mironova V. , Zelikhina S. , Korennoy F. , Grishchenko M. , and Carvalho M. S. , Spatial Patterns of West Nile Virus Distribution in the Volgograd Region of Russia, A Territory With Long-Existing Foci, PLoS Neglected Tropical Diseases. (2022) 16, no. 1, 10.1371/journal.pntd.0010145, e0010145. 35100289 PMC8803152

[68] Redding D. W. , Atkinson P. M. , and Cunningham A. A. , et al.Impacts of Environmental and Socio-Economic Factors on Emergence and Epidemic Potential of Ebola in Africa, Nature Communications. (2019) 10, no. 1, 10.1038/s41467-019-12499-6, 2-s2.0-85073408117, 4531. PMC6794280 31615986

[69] Mursel S. , Alter N. , and Slavit L. , et al.Estimation of Ebola’s Spillover Infection Exposure in Sierra Leone Based on Sociodemographic and Economic Factors, PLoS ONE. (2022) 17, no. 9, 10.1371/journal.pone.0271886, e0271886. 36048780 PMC9436100

[70] Shapiro J. T. , Sovie A. R. , Faller C. R. , Monadjem A. , Fletcher R. J.Jr., and McCleery R. A. , Ebola Spillover Correlates With Bat Diversity, European Journal of Wildlife Research. (2020) 66, no. 1, 10.1007/s10344-019-1346-7, 12.

[71] Keyel A. C. , Timm O. E. , and Backenson P. B. , et al.Seasonal Temperatures and Hydrological Conditions Improve the Prediction of West Nile Virus Infection Rates in Culex Mosquitoes and Human Case Counts in New York and Connecticut, PLoS ONE. (2019) 14, no. 6, 10.1371/journal.pone.0217854, 2-s2.0-85066609076, e0217854. 31158250 PMC6546252

[72] Sallam M. F. , Michaels S. R. , and Riegel C. , et al.Spatio-Temporal Distribution of Vector-Host Contact (VHC) Ratios and Ecological Niche Modeling of the West Nile Virus Mosquito Vector, *Culex quinquefasciatus*, in the City of New Orleans, LA, USA, International Journal of Environmental Research and Public Health. (2017) 14, no. 8, 10.3390/ijerph14080892, 2-s2.0-85027067382, 892. 28786934 PMC5580596

[73] Cunningham A. A. , Daszak P. , and Wood J. L. N. , One Health, Emerging Infectious Diseases and Wildlife: Two Decades of Progress?, Philosophical Transactions of the Royal Society B: Biological Sciences. (2017) 372, no. 1725, 10.1098/rstb.2016.0167, 2-s2.0-85020459691, 20160167. PMC5468692 28584175

[74] Kelly T. R. , Karesh W. B. , and Johnson C. K. , et al.One Health Proof of Concept: Bringing a Transdisciplinary Approach to Surveillance for Zoonotic Viruses at the Human–Wild Animal Interface, Preventive Veterinary Medicine. (2017) 137, 112–118, 10.1016/j.prevetmed.2016.11.023, 2-s2.0-85009215288. 28034593 PMC7132382

[75] Roster K. , Connaughton C. , and Rodrigues F. A. , Forecasting New Diseases in Low-Data Settings Using Transfer Learning, Chaos, Solitons & Fractals. (2022) 161, 10.1016/j.chaos.2022.112306, 112306. 35765601 PMC9222348

[76] Jagadesh S. , Zhao C. , Mulchandani R. , and Van Boeckel T. P. , Mapping Global Bushmeat Activities to Improve Zoonotic Spillover Surveillance by Using Geospatial Modeling, Emerging Infectious Diseases. (2023) 29, no. 4, 742–750, 10.3201/eid2904.221022. 36957996 PMC10045693

[77] Kawasaki J. , Suzuki T. , and Hamada M. , Hidden Challenges in Evaluating Spillover Risk of Zoonotic Viruses Using Machine Learning Models, Communications Medicine. (2025) 5, no. 1, 10.1038/s43856-025-00903-w, 187. 40394176 PMC12092720

[78] Allen T. , Murray K. A. , and Zambrana-Torrelio C. , et al.Global Hotspots and Correlates of Emerging Zoonotic Diseases, Nature Communications. (2017) 8, no. 1, 10.1038/s41467-017-00923-8, 2-s2.0-85032192849, 1124. PMC5654761 29066781

[79] Mukherjee D. , Sagar K. , and Kobialka R. M. , et al.Filling the Gap: Artificial Intelligence-Driven One Health Integration to Strengthen Pandemic Preparedness in Resource-Limited Settings, Frontiers in Public Health. (2025) 13, 10.3389/fpubh.2025.1707306, 1707306. 41450491 PMC12727988

[80] Meisner J. , Baines A. , and Ngere I. , et al.Mapping Hotspots of Zoonotic Pathogen Emergence: An Integrated Model-Based and Participatory-Based Approach, The Lancet Planetary Health. (2025) 9, no. 1, e14–e22, 10.1016/S2542-5196(24)00309-7. 39855227

[81] Astbury C. C. , Lee K. M. , and McLeod R. , et al.Policies to Prevent Zoonotic Spillover: A Systematic Scoping Review of Evaluative Evidence, Globalization and Health. (2023) 19, no. 1, 10.1186/s12992-023-00986-x, 82. 37940941 PMC10634115

[82] Eby P. , Peel A. J. , and Hoegh A. , et al.Pathogen Spillover Driven by Rapid Changes in Bat Ecology, Nature. (2023) 613, no. 7943, 340–344, 10.1038/s41586-022-05506-2. 36384167 PMC9768785

[83] Probert W. J. M. , Lakkur S. , and Fonnesbeck C. J. , et al.Context Matters: Using Reinforcement Learning to Develop Human-Readable, State-Dependent Outbreak Response Policies, Philosophical Transactions of the Royal Society B: Biological Sciences. (2019) 374, no. 1776, 10.1098/rstb.2018.0277, 2-s2.0-85065400140, 20180277. PMC6558555 31104604

[84] Lepenioti K. , Bousdekis A. , Apostolou D. , and Mentzas G. , Prescriptive Analytics: Literature Review and Research Challenges, International Journal of Information Management. (2020) 50, 57–70, 10.1016/j.ijinfomgt.2019.04.003.

[85] Menezes B. C. , Kelly J. D. , Leal A. G. , and Le Roux G. C. , Predictive, Prescriptive and Detective Analytics for Smart Manufacturing in the Information Age, IFAC-PapersOnLine. (2019) 52, no. 1, 568–573, 10.1016/j.ifacol.2019.06.123, 2-s2.0-85070567506.

[86] Bastani H. , Drakopoulos K. , and Gupta V. , et al.Efficient and Targeted COVID-19 Border Testing via Reinforcement Learning, Nature. (2021) 599, no. 7883, 108–113, 10.1038/s41586-021-04014-z. 34551425

[87] Judson S. D. and Dowdy D. W. , Modeling Zoonotic and Vector-Borne Viruses, Current Opinion in Virology. (2024) 67, 10.1016/j.coviro.2024.101428, 101428. 39047313 PMC11292992

[88] Tonelli A. , Blagrove M. S. C. , Wardeh M. , and Di Marco M. , A Framework to Predict Zoonotic Hosts Under Data Uncertainty: A Case Study on Betacoronaviruses, Methods in Ecology and Evolution. (2025) 16, no. 3, 611–624, 10.1111/2041-210X.14500.

[89] Zwiers L. C. , Grobbee D. E. , Uijl A. , and Ong D. S. Y. , Federated Learning as a Smart Tool for Research on Infectious Diseases, BMC Infectious Diseases. (2024) 24, no. 1, 10.1186/s12879-024-10230-5, 1327. 39573994 PMC11580691

[90] Tsueng G. , Cano M. A. A. , and Bento J. , et al.Developing a Standardized but Extendable Framework to Increase the Findability of Infectious Disease Datasets, Scientific Data. (2023) 10, no. 1, 10.1038/s41597-023-01968-9, 99. 36823157 PMC9950378

[91] Shanbehzadeh M. , Nopour R. , and Kazemi-Arpanahi H. , Designing a Standardized Framework for Data Integration Between Zoonotic Diseases Systems: Towards One Health Surveillance, Informatics in Medicine Unlocked. (2022) 30, 10.1016/j.imu.2022.100893, 100893.

[92] Becker D. J. , Albery G. F. , and Sjodin A. R. , et al.Optimising Predictive Models to Prioritise Viral Discovery in Zoonotic Reservoirs, The Lancet Microbe. (2022) 3, no. 8, e625–e637, 10.1016/S2666-5247(21)00245-7. 35036970 PMC8747432

[93] Villanueva-Miranda I. , Xiao G. , and Xie Y. , Artificial Intelligence in Early Warning Systems for Infectious Disease Surveillance: A Systematic Review, Frontiers in Public Health. (2025) 13, 10.3389/fpubh.2025.1609615, 1609615. 40626156 PMC12230060

[94] Moccia C. , Moirano G. , and Popovic M. , et al.Machine Learning in Causal Inference for Epidemiology, European Journal of Epidemiology. (2024) 39, no. 10, 1097–1108, 10.1007/s10654-024-01173-x. 39535572 PMC11599438

[95] Du H. , Zhao Y. , and Zhao J. , et al.Advancing Real-Time Infectious Disease Forecasting Using Large Language Models, Nature Computational Science. (2025) 5, no. 6, 467–480, 10.1038/s43588-025-00798-6. 40481184

[96] Kraemer M. U. G. , Tsui J. L. H. , and Chang S. Y. , et al.Artificial Intelligence for Modelling Infectious Disease Epidemics, Nature. (2025) 638, no. 8051, 623–635, 10.1038/s41586-024-08564-w. 39972226 PMC11987553

[97] Roberts M. , Dobson A. , Restif O. , and Wells K. , Challenges in Modelling the Dynamics of Infectious Diseases at the Wildlife-Human Interface, Epidemics. (2021) 37, 10.1016/j.epidem.2021.100523, 100523. 34856500 PMC8603269

