# Supplementary material for: A Scoping Review of Machine Learning Applications Across Epidemiological Stages of Zoonotic Disease
Source: Transbound Emerg Dis. 2026 May 14;2026:2215823. doi: 10.1155/tbed/2215823 (PMC13176625; doi:10.1155/tbed/2215823)
Supplement: Supplementary file 1 — Supporting Information Text S1: Search strategies for databases of publications. Figure S1: Flow diagram of the selection procedure. Figure S2: Data types and machine learning models applied to management in human populations. Figure S3: Data types and machine learning models applied to management in animal reservoirs and vectors. (A) Chord diagram showing the distribution of ML models across the functional domains of diagnosis, epidemiology, and intervention. The numbers indicate the total count of model applications within each application domain. (B) Chord diagram showing the distribution of data types across the same functional domains. The numbers indicate the total count of applications of the data types within each domain. Figure S4: Data types and machine learning models applied to management in animal–human interface. (A) Chord diagram showing the distribution of ML models across the functional domains of diagnosis, epidemiology, and intervention. The numbers indicate the total count of model applications within each application domain. (B) Chord diagram showing the distribution of data types across the same functional domains. The numbers indicate the total count of applications of the data types within each domain. Table S1: Epidemiological characteristics of the zoonotic diseases analyzed in this scoping review. Table S2: Summary of algorithm–data type coapplications organized by epidemiological stage and functional domain. Table S3: Representative borderline cases with prediction targets, assigned stages, and classification rationale. [file TBED-2026-2215823-s001.docx]

**Supplementary Information**

**A scoping review of machine learning applications across** **epidemiological stages of zoonotic disease**

Yinsheng Zhang^1,2,3^, Yifan Sun^1^, Jinchen Wang^1^, Luqi Wang^1^, Ruying Fang^1,2,3^, Xiaolong Wu^1^, Xin Yang^1^, Yiyang Guo^1^, Sen Li^1^*

1.School of Environmental Science and Engineering, Huazhong University of Science and Technology, Wuhan 430074, Hubei, P.R. China

2.Institute of Artificial Intelligence, Huazhong University of Science and Technology, Wuhan 430074, Hubei, P.R. China

3.School of Artificial Intelligence and Automation, Huazhong University of Science and Technology, Wuhan, 430074, Hubei, P.R. China

4. Earth and Life Institute, Université Catholique de Louvain (UCLouvain), Louvain-la-Neuve, B1348, Belgium

**^*^Corresponding author**

Sen Li (senli@hust.edu.cn)

**Supplementary Text**

**Text S1 Search strategies for databases of publications**

**Web of Science Search Strategy**

1. TS = ((“avian influenza” OR “bird flu” OR malaria OR nipah OR “middle East respiratory syndrome” OR “MERS-CoV” OR ebola OR zika virus OR “West Nile” OR “chikungunya” OR “rift Valley fever” OR “H1N1” OR “monkeypox” OR “severe acute respiratory syndrome coronavirus” OR “SARS-COV” OR “SARS coronavirus” OR COVID-19 OR “SARS-COV-2” OR “human immunodeficiency virus” OR Lassa))

2. TS= ((“artificial intelligence” OR “machine learning” OR “deep learning” OR “reinforcement learning” OR “natural language processing”))

3. TS= ((“identification” OR “recognition” OR “screen*” OR “diagno*” OR “early warning” OR detection OR monitor OR surveillance))

4. TS= ((gender OR age OR race OR clinic* OR animal))

5. TS= #4 AND #3 AND #2 AND #1

6. TS= ((pandemic* OR epidemi* OR endemic* OR outbreak* OR epizootic* OR enzootic* OR panzootic*OR zoono*))

7. TS= ((transmission OR transmit* OR spread* OR propagat*))

8. TS= #6 AND #7 AND #2 AND #1

9. TS= ((control* OR decision OR intervention OR prescriptive OR policy OR strateg*))

10. TS= #9 AND #2 AND #1

**PubMed Search Strategy**

1. All Fields: ((“avian influenza” OR “bird flu” OR malaria OR nipah OR “middle East respiratory syndrome” OR “MERS-CoV” OR ebola OR zika virus OR “West Nile” OR “chikungunya” OR “rift Valley fever” OR “H1N1” OR “monkeypox” OR “severe acute respiratory syndrome coronavirus” OR “SARS-COV” OR “SARS coronavirus” OR COVID-19 OR “SARS-COV-2” OR “human immunodeficiency virus” OR Lassa))

2. All Fields: ((“artificial intelligence” OR “machine learning” OR “deep learning” OR “reinforcement learning” OR “natural language processing”))

3. All Fields: ((“identification” OR “recognition” OR “screen*” OR “early diagno*” OR “early warning” OR detection OR monitor OR surveillance))

4. All Fields: ((gender OR age OR race OR clinic* OR animal))

5. All Fields: #4 AND #3 AND #2 AND #1

6. All Fields: ((pandemic* OR epidemi* OR endemic* OR outbreak* OR epizootic* OR enzootic* OR panzootic*OR zoono*))

7. All Fields: ((transmission OR transmit* OR spread* OR propagat*))

8. All Fields: #6 AND #7 AND #2 AND #1

9. All Fields: ((control* OR decision OR intervention OR prescriptive OR policy OR strateg*))

10. All Fields: #9 AND #2 AND #1

**Supplementary Figures**


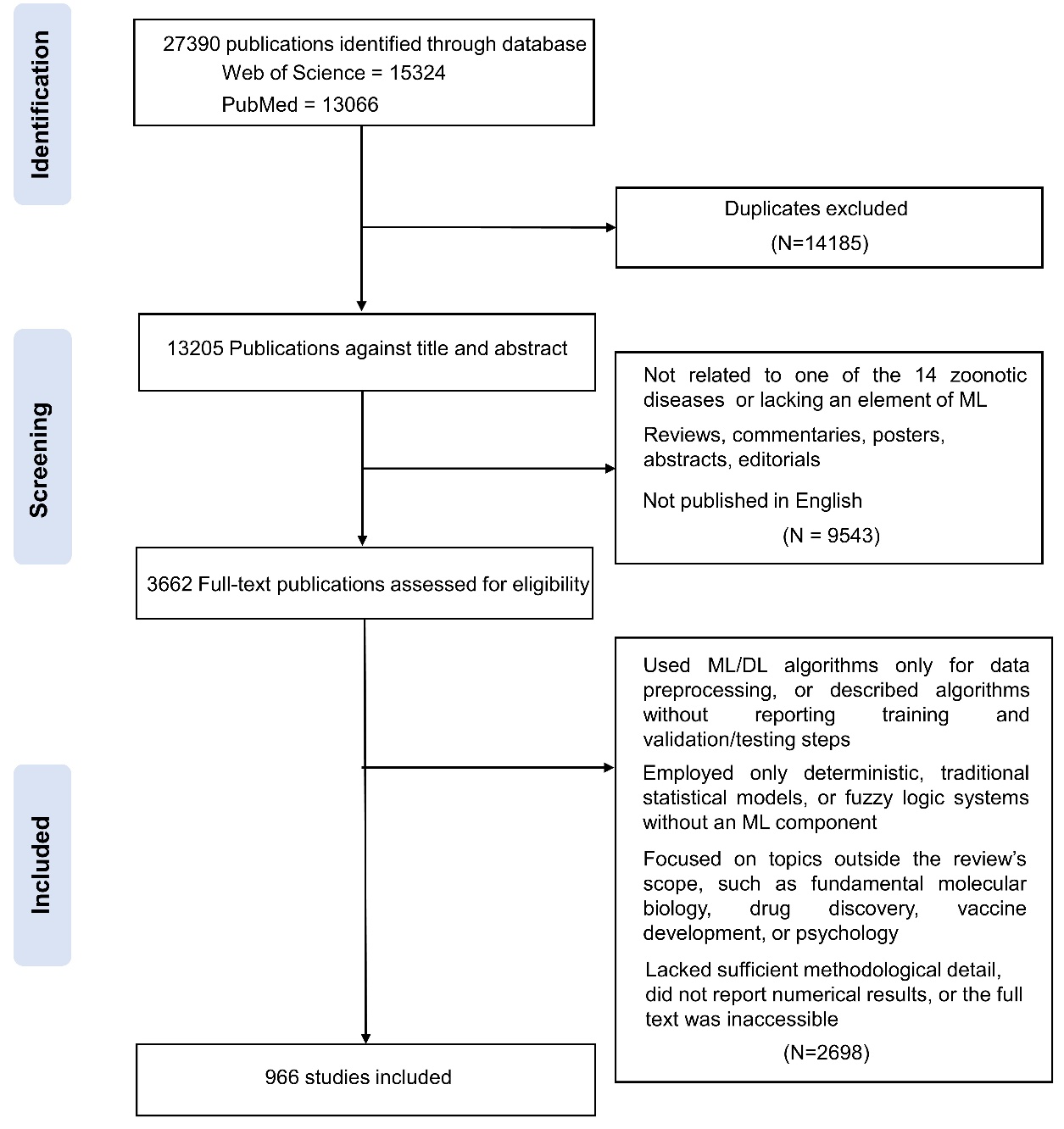


**Fig. S1 Flow diagram of the selection procedure**


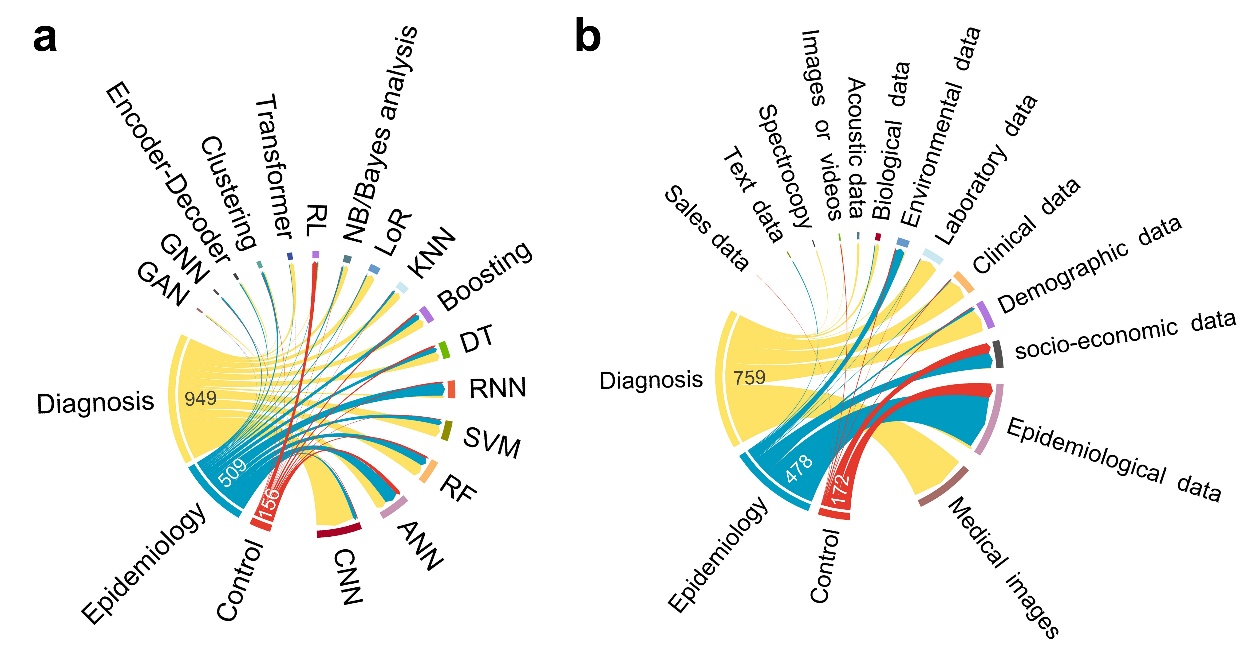


**Fig. S2 Data types and machine Learning models applied to management in human populations**

(A) Chord diagram showing the distribution of ML models across the functional domains of Diagnosis, Epidemiology, and Intervention. The numbers indicate the total count of model applications within each application domain. (B) Chord diagram showing the distribution of data types across the same functional domains. The numbers indicate the total count of applications of the data types within each domain.


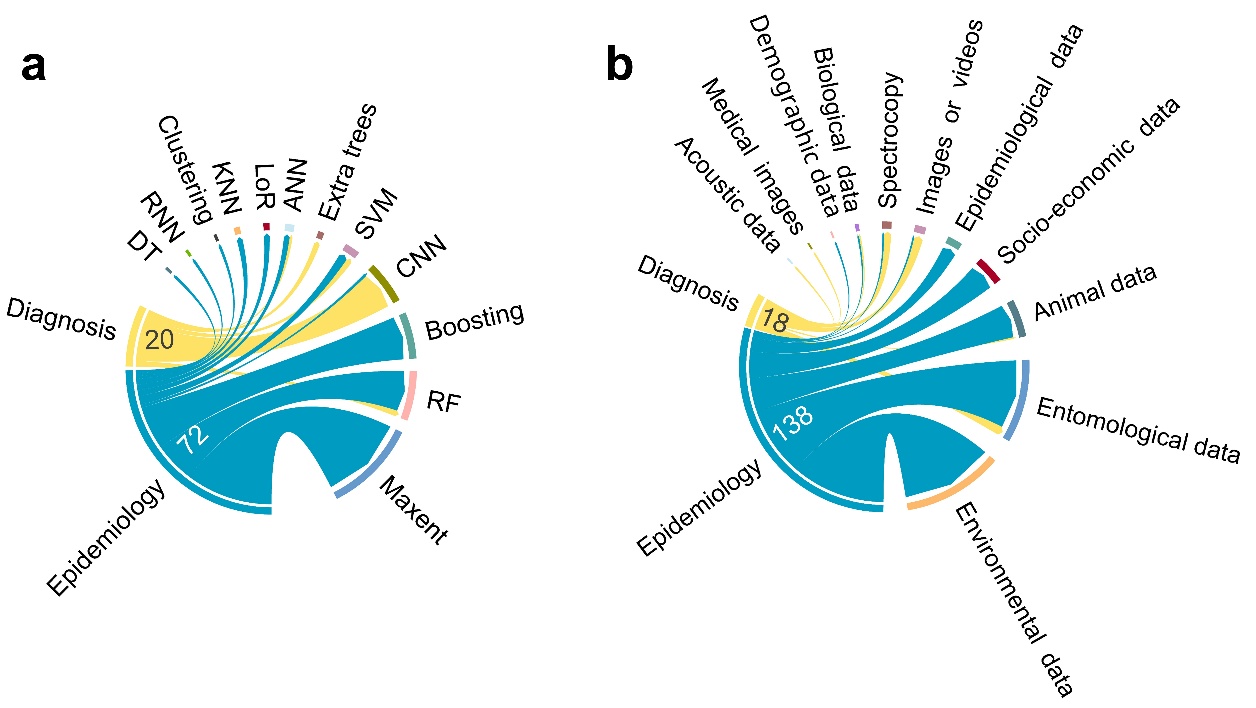


**Fig. S3 Data types and machine Learning models applied to management in animal reservoirs and vectors**

(A) Chord diagram showing the distribution of ML models across the functional domains of Diagnosis, Epidemiology, and Intervention. The numbers indicate the total count of model applications within each application domain. (B) Chord diagram showing the distribution of data types across across the same functional domains. The numbers indicate the total count of applications of the data types within each domain.


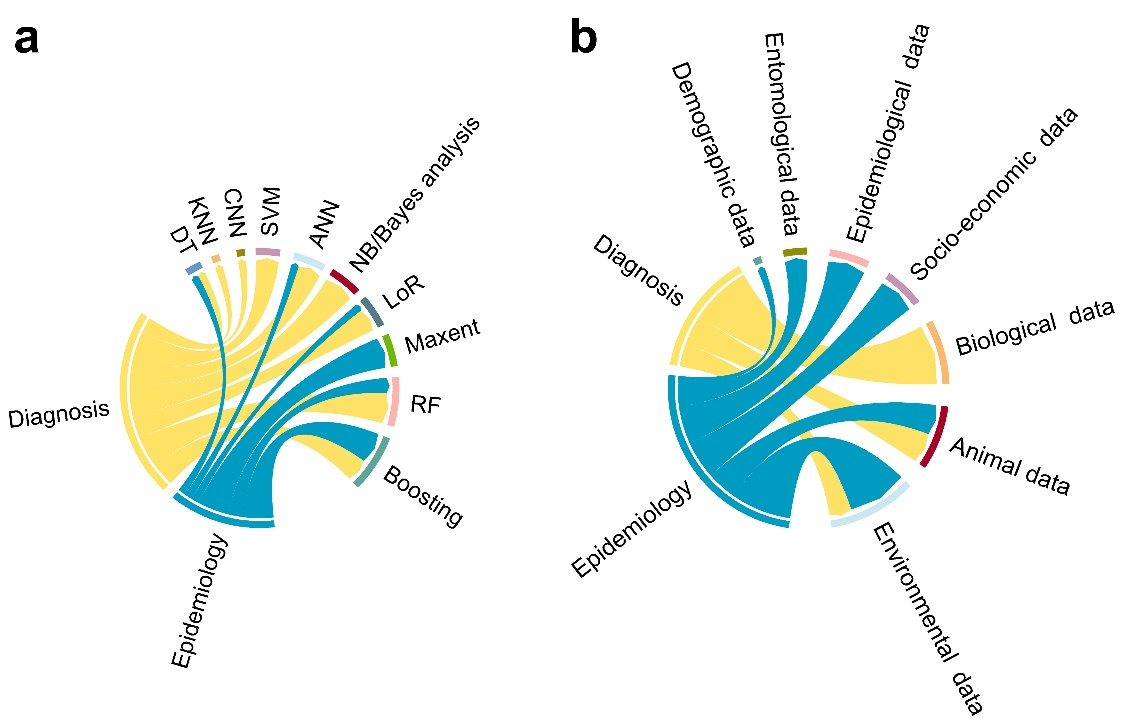


**Fig. S4 Data types and machine Learning models applied to management in animal-human interface**

(A) Chord diagram showing the distribution of ML models across the functional domains of Diagnosis, Epidemiology, and Intervention. The numbers indicate the total count of model applications within each application domain. (B) Chord diagram showing the distribution of data types across across the same functional domains. The numbers indicate the total count of applications of the data types within each domain.

**Supplementary Table**

**Table S1** Epidemiological characteristics of the zoonotic diseases analyzed in this scoping review

| Transmission mode | Zoonotic disease | Pathogens | Primary animal reservoir or host | Primary transmission route | Human-human transmission |
| --- | --- | --- | --- | --- | --- |
| Direct contact | HIV | Human Immunodeficiency Virus | Chimpanzees, Sooty Mangabeys | Contact with infected primate bodily fluids (e.g., during hunting/butchering). | Yes |
|  | Ebola disease | Ebolavirus genus | Fruit bats, Apes, Monkeys | Direct contact with the blood, secretions, or organs of infected animals. | Yes |
|  | Monkeypox | Monkeypox virus | Rodents, Primates | Direct contact with lesions, body fluids, or contaminated materials from infected animals. | Yes |
|  | Nipah infection | Nipah virus | Pigs, dogs, goats,  cats, horses,  sheep and bats | Direct  transmission  from an infected  animal | Yes |
|  | Lassa fever | Lassa mammarenavirus | Rodents | Contact with food, water, or surfaces contaminated with the urine or feces of infected rats. | Yes |
| Airborne | COVID-19 | SARS-CoV-2 | Bats (probable), with potential intermediate hosts | Inhalation of droplets/aerosols from infected animals or contact with contaminated surfaces. | Yes |
|  | H1N1 influenza | Influenza A (H1N1) virus | Aquatic waterfowl, Pigs | Inhalation of droplets from infected animals (pigs, birds) or contact with contaminated environments. | Yes |
|  | HPAI | HPAI virus (e.g., H5N1) | Wild waterfowl, Poultry | Direct, close contact with infected birds (live or dead) or their contaminated environments. | Rare |
|  | MERS | MERS-CoV | Dromedary camels | Close contact with infected dromedary camels (e.g., consumption of raw milk/meat, contact with secretions). | Yes |
| Vector-borne | Malaria | *Plasmodium species* | Mosquitoes (Anopheles), Non-human primates | Bite of an infected female Anopheles mosquito. | No |
|  | Chikungunya | Chikungunya virus (CHIKV) | Mosquitoes (Aedes), Monkeys | Bite of infected mosquito. | No |
|  | Rift Valley fever (RVF) | RVF virus | Domestic animals, Mosquitoes | Bite of infected mosquitoes; contact with blood/organs of infected livestock. | No |
|  | West Nile fever | West Nile virus | Birds, Mosquitoes | Bite of an infected mosquito. | No |
|  | Zika virus disease | Zika virus (ZIKV) | Primates, Mosquitoes (Aedes) | Bite of an infected mosquito. | Yes |

**Table S2** Summary of algorithm-data type co-applications by epidemiological stage and functional domain

| **Application Scenario** | **Algorithm** | **Data Type** | **No. of Papers** |
| --- | --- | --- | --- |
| Human Populations - Diagnosis | CNN | Medical Images | 268 |
|  | RF | Demographic Data | 60 |
|  | RF | Clinical Data | 54 |
|  | Boosting | Demographic Data | 51 |
|  | Boosting | Clinical Data | 45 |
|  | LoR | Demographic Data | 44 |
|  | RF | Laboratory Data | 44 |
|  | DT | Demographic Data | 41 |
|  | SVM | Laboratory Data | 41 |
|  | SVM | Demographic Data | 40 |
|  | Boosting | Laboratory Data | 40 |
|  | DT | Clinical Data | 36 |
|  | ANN | Demographic Data | 35 |
|  | LoR | Clinical Data | 35 |
|  | SVM | Clinical Data | 34 |
|  | ANN | Laboratory Data | 34 |
|  | ANN | Clinical Data | 32 |
|  | LoR | Laboratory Data | 31 |
|  | DT | Laboratory Data | 31 |
|  | SVM | Medical Images | 30 |
|  | ANN | Medical Images | 29 |
|  | Transformer | Medical Images | 28 |
|  | KNN | Demographic Data | 27 |
|  | RF | Medical Images | 24 |
|  | NB/Bayes analysis | Demographic Data | 23 |
|  | KNN | Clinical Data | 23 |
|  | KNN | Laboratory Data | 21 |
|  | NB/Bayes analysis | Clinical Data | 18 |
|  | NB/Bayes analysis | Laboratory Data | 17 |
|  | KNN | Medical Images | 17 |
|  | CNN | Demographic Data | 16 |
|  | CNN | Clinical Data | 16 |
|  | CNN | Laboratory Data | 14 |
|  | GAN | Medical Images | 11 |
|  | RNN | Medical Images | 11 |
|  | RF | Biological Data | 10 |
|  | LoR | Medical Images | 10 |
|  | NB/Bayes analysis | Medical Images | 10 |
|  | DT | Medical Images | 10 |
|  | Boosting | Medical Images | 9 |
|  | SVM | Biological Data | 8 |
|  | CNN | Acoustic Data | 8 |
|  | Reinforcement learning | Medical Images | 7 |
|  | Clustering | Demographic Data | 6 |
|  | LoR | Biological Data | 6 |
|  | DT | Biological Data | 6 |
|  | Encoder-Decoder | Medical Images | 6 |
|  | Clustering | Clinical Data | 5 |
|  | RNN | Laboratory Data | 5 |
|  | RF | Epidemiological Data | 5 |
|  | KNN | Epidemiological Data | 5 |
|  | ANN | Epidemiological Data | 5 |
|  | DT | Epidemiological Data | 5 |
|  | KNN | Biological Data | 5 |
|  | ANN | Biological Data | 5 |
|  | Boosting | Epidemiological Data | 4 |
|  | SVM | Epidemiological Data | 4 |
|  | LoR | Epidemiological Data | 4 |
|  | CNN | Biological Data | 4 |
|  | NB/Bayes analysis | Biological Data | 4 |
|  | SVM | Spectrocopy | 4 |
|  | Encoder-Decoder | Demographic Data | 3 |
|  | RNN | Clinical Data | 3 |
|  | Clustering | Laboratory Data | 3 |
|  | NB/Bayes analysis | Epidemiological Data | 3 |
|  | ANN | Environmental Data | 3 |
|  | SVM | Acoustic Data | 3 |
|  | CNN | Images Or Videos | 3 |
|  | RNN | Demographic Data | 2 |
|  | Transformer | Clinical Data | 2 |
|  | Encoder-Decoder | Laboratory Data | 2 |
|  | Transformer | Laboratory Data | 2 |
|  | CNN | Epidemiological Data | 2 |
|  | Boosting | Biological Data | 2 |
|  | Clustering | Biological Data | 2 |
|  | Clustering | Medical Images | 2 |
|  | DT | Acoustic Data | 2 |
|  | KNN | Spectrocopy | 2 |
|  | ANN | Spectrocopy | 2 |
|  | LoR | Spectrocopy | 2 |
|  | Transformer | Demographic Data | 1 |
|  | Encoder-Decoder | Clinical Data | 1 |
|  | RNN | Epidemiological Data | 1 |
|  | Transformer | Epidemiological Data | 1 |
|  | Clustering | Epidemiological Data | 1 |
|  | Boosting | Text Data | 1 |
|  | RF | Text Data | 1 |
|  | SVM | Text Data | 1 |
|  | LoR | Text Data | 1 |
|  | NB/Bayes analysis | Text Data | 1 |
|  | DT | Text Data | 1 |
|  | Encoder-Decoder | Biological Data | 1 |
|  | RNN | Biological Data | 1 |
|  | Transformer | Biological Data | 1 |
|  | RF | Environmental Data | 1 |
|  | SVM | Environmental Data | 1 |
|  | LoR | Environmental Data | 1 |
|  | DT | Environmental Data | 1 |
|  | Encoder-Decoder | Environmental Data | 1 |
|  | RF | Acoustic Data | 1 |
|  | KNN | Acoustic Data | 1 |
|  | ANN | Acoustic Data | 1 |
|  | LoR | Acoustic Data | 1 |
|  | NB/Bayes analysis | Acoustic Data | 1 |
|  | RNN | Acoustic Data | 1 |
|  | Boosting | Socio Economic Data | 1 |
|  | KNN | Socio Economic Data | 1 |
|  | Transformer | Socio Economic Data | 1 |
|  | Boosting | Spectrocopy | 1 |
|  | RF | Spectrocopy | 1 |
|  | NB/Bayes analysis | Spectrocopy | 1 |
| Human Populations - Epidemiology | ANN | Epidemiological Data | 101 |
|  | RNN | Epidemiological Data | 97 |
|  | SVM | Epidemiological Data | 42 |
|  | RF | Epidemiological Data | 41 |
|  | Boosting | Epidemiological Data | 40 |
|  | DT | Epidemiological Data | 39 |
|  | RF | Socio Economic Data | 28 |
|  | CNN | Epidemiological Data | 27 |
|  | RF | Environmental Data | 27 |
|  | ANN | Socio Economic Data | 24 |
|  | Boosting | Socio Economic Data | 23 |
|  | DT | Socio Economic Data | 21 |
|  | RNN | Socio Economic Data | 20 |
|  | Boosting | Environmental Data | 19 |
|  | KNN | Epidemiological Data | 18 |
|  | Clustering | Epidemiological Data | 17 |
|  | ANN | Environmental Data | 16 |
|  | NB/Bayes analysis | Epidemiological Data | 11 |
|  | GNN | Epidemiological Data | 11 |
|  | DT | Environmental Data | 11 |
|  | Encoder-Decoder | Epidemiological Data | 10 |
|  | Clustering | Socio Economic Data | 8 |
|  | Boosting | Demographic Data | 7 |
|  | LoR | Epidemiological Data | 7 |
|  | KNN | Environmental Data | 7 |
|  | SVM | Environmental Data | 7 |
|  | CNN | Socio Economic Data | 7 |
|  | GNN | Socio Economic Data | 7 |
|  | RF | Demographic Data | 6 |
|  | ANN | Demographic Data | 6 |
|  | KNN | Socio Economic Data | 6 |
|  | Encoder-Decoder | Socio Economic Data | 6 |
|  | Reinforcement learning | Epidemiological Data | 5 |
|  | SVM | Socio Economic Data | 5 |
|  | Transformer | Epidemiological Data | 4 |
|  | SVM | Demographic Data | 3 |
|  | LoR | Socio Economic Data | 3 |
|  | NB/Bayes analysis | Socio Economic Data | 3 |
|  | KNN | Demographic Data | 2 |
|  | DT | Demographic Data | 2 |
|  | Clustering | Demographic Data | 2 |
|  | GAN | Epidemiological Data | 2 |
|  | ANN | Text Data | 2 |
|  | ANN | Biological Data | 2 |
|  | CNN | Environmental Data | 2 |
|  | NB/Bayes analysis | Environmental Data | 2 |
|  | RNN | Environmental Data | 2 |
|  | Reinforcement learning | Socio Economic Data | 2 |
|  | CNN | Demographic Data | 1 |
|  | LoR | Demographic Data | 1 |
|  | NB/Bayes analysis | Demographic Data | 1 |
|  | Boosting | Clinical Data | 1 |
|  | RF | Clinical Data | 1 |
|  | KNN | Clinical Data | 1 |
|  | SVM | Clinical Data | 1 |
|  | DT | Clinical Data | 1 |
|  | RNN | Clinical Data | 1 |
|  | Boosting | Laboratory Data | 1 |
|  | KNN | Laboratory Data | 1 |
|  | ANN | Laboratory Data | 1 |
|  | DT | Laboratory Data | 1 |
|  | RNN | Laboratory Data | 1 |
|  | SVM | Text Data | 1 |
|  | RNN | Text Data | 1 |
|  | Clustering | Text Data | 1 |
|  | RF | Biological Data | 1 |
|  | CNN | Biological Data | 1 |
|  | NB/Bayes analysis | Biological Data | 1 |
|  | RNN | Biological Data | 1 |
|  | Clustering | Biological Data | 1 |
|  | GNN | Biological Data | 1 |
|  | LoR | Environmental Data | 1 |
|  | Clustering | Environmental Data | 1 |
|  | GAN | Socio Economic Data | 1 |
|  | Transformer | Socio Economic Data | 1 |
| Human Populations - Intervention | Reinforcement learning | Epidemiological Data | 32 |
|  | ANN | Epidemiological Data | 28 |
|  | Reinforcement learning | Socio Economic Data | 28 |
|  | DT | Epidemiological Data | 13 |
|  | RF | Epidemiological Data | 12 |
|  | ANN | Socio Economic Data | 12 |
|  | Boosting | Epidemiological Data | 10 |
|  | RNN | Epidemiological Data | 9 |
|  | RF | Socio Economic Data | 9 |
|  | DT | Socio Economic Data | 9 |
|  | RNN | Socio Economic Data | 8 |
|  | Boosting | Socio Economic Data | 7 |
|  | CNN | Epidemiological Data | 6 |
|  | RF | Clinical Data | 4 |
|  | SVM | Epidemiological Data | 4 |
|  | CNN | Socio Economic Data | 4 |
|  | Boosting | Demographic Data | 3 |
|  | RF | Demographic Data | 3 |
|  | Reinforcement learning | Demographic Data | 3 |
|  | ANN | Clinical Data | 3 |
|  | RF | Environmental Data | 3 |
|  | ANN | Demographic Data | 2 |
|  | NB/Bayes analysis | Demographic Data | 2 |
|  | DT | Demographic Data | 2 |
|  | Clustering | Demographic Data | 2 |
|  | Boosting | Clinical Data | 2 |
|  | Clustering | Clinical Data | 2 |
|  | KNN | Epidemiological Data | 2 |
|  | Clustering | Epidemiological Data | 2 |
|  | Boosting | Environmental Data | 2 |
|  | ANN | Environmental Data | 2 |
|  | DT | Environmental Data | 2 |
|  | Reinforcement learning | Environmental Data | 2 |
|  | SVM | Socio Economic Data | 2 |
|  | GNN | Socio Economic Data | 2 |
|  | ANN | Sales Data | 2 |
|  | SVM | Demographic Data | 1 |
|  | LoR | Demographic Data | 1 |
|  | GNN | Demographic Data | 1 |
|  | SVM | Clinical Data | 1 |
|  | LoR | Clinical Data | 1 |
|  | DT | Clinical Data | 1 |
|  | Reinforcement learning | Clinical Data | 1 |
|  | GNN | Clinical Data | 1 |
|  | Boosting | Laboratory Data | 1 |
|  | NB/Bayes analysis | Laboratory Data | 1 |
|  | Reinforcement learning | Laboratory Data | 1 |
|  | LoR | Epidemiological Data | 1 |
|  | NB/Bayes analysis | Epidemiological Data | 1 |
|  | Transformer | Epidemiological Data | 1 |
|  | GNN | Epidemiological Data | 1 |
|  | ELM | Epidemiological Data | 1 |
|  | Extra Trees | Epidemiological Data | 1 |
|  | LoR | Environmental Data | 1 |
|  | NB/Bayes analysis | Environmental Data | 1 |
|  | Clustering | Environmental Data | 1 |
|  | KNN | Socio Economic Data | 1 |
|  | LoR | Socio Economic Data | 1 |
|  | NB/Bayes analysis | Socio Economic Data | 1 |
|  | Transformer | Socio Economic Data | 1 |
|  | Clustering | Socio Economic Data | 1 |
|  | Boosting | Sales Data | 1 |
|  | SVM | Sales Data | 1 |
|  | NB/Bayes analysis | Sales Data | 1 |
|  | DT | Sales Data | 1 |
|  | ELM | Sales Data | 1 |
|  | Extra Trees | Sales Data | 1 |
| Animal-Human Interface - Diagnosis | RF | Biological Data | 5 |
|  | ANN | Biological Data | 3 |
|  | SVM | Biological Data | 3 |
|  | LoR | Biological Data | 3 |
|  | NB/Bayes analysis | Biological Data | 3 |
|  | Boosting | Animal Data | 2 |
|  | Boosting | Environmental Data | 1 |
|  | RF | Environmental Data | 1 |
|  | NB/Bayes analysis | Environmental Data | 1 |
|  | Boosting | Biological Data | 1 |
|  | KNN | Biological Data | 1 |
|  | DT | Biological Data | 1 |
|  | Boosting | Entomological Data | 1 |
|  | RF | Animal Data | 1 |
|  | NB/Bayes analysis | Animal Data | 1 |
| Animal-Human Interface - Epidemiology | Boosting | Environmental Data | 4 |
|  | MaxEnt | Environmental Data | 4 |
|  | MaxEnt | Socio Economic Data | 3 |
|  | MaxEnt | Epidemiological Data | 3 |
|  | Boosting | Animal Data | 3 |
|  | RF | Environmental Data | 2 |
|  | Boosting | Socio Economic Data | 2 |
|  | Boosting | Epidemiological Data | 2 |
|  | RF | Epidemiological Data | 2 |
|  | MaxEnt | Entomological Data | 2 |
|  | MaxEnt | Animal Data | 2 |
|  | LoR | Demographic Data | 1 |
|  | ANN | Environmental Data | 1 |
|  | LoR | Environmental Data | 1 |
|  | ANN | Socio Economic Data | 1 |
|  | RF | Entomological Data | 1 |
|  | RF | Animal Data | 1 |
|  | ANN | Animal Data | 1 |
| Animal Reservoirs - Diagnosis | CNN | Images Or Videos | 9 |
|  | CNN | Entomological Data | 5 |
|  | CNN | Spectrocopy | 3 |
|  | Extra trees | Images Or Videos | 2 |
|  | RF | Biological Data | 1 |
|  | CNN | Acoustic Data | 1 |
|  | SVM | Images Or Videos | 1 |
|  | CNN | Medical Images | 1 |
|  | RF | Animal Data | 1 |
|  | ANN | Spectrocopy | 1 |
|  | SVM | Spectrocopy | 1 |
| Animal Reservoirs - Epidemiology | MaxEnt | Environmental Data | 30 |
|  | MaxEnt | Entomological Data | 25 |
|  | Boosting | Environmental Data | 15 |
|  | RF | Environmental Data | 14 |
|  | RF | Entomological Data | 9 |
|  | Boosting | Animal Data | 9 |
|  | MaxEnt | Animal Data | 8 |
|  | MaxEnt | Socio Economic Data | 7 |
|  | Boosting | Entomological Data | 7 |
|  | MaxEnt | Epidemiological Data | 5 |
|  | Boosting | Socio Economic Data | 5 |
|  | RF | Socio Economic Data | 5 |
|  | RF | Epidemiological Data | 3 |
|  | RF | Animal Data | 3 |
|  | Boosting | Epidemiological Data | 2 |
|  | ANN | Environmental Data | 2 |
|  | SVM | Environmental Data | 2 |
|  | LoR | Environmental Data | 2 |
|  | SVM | Entomological Data | 2 |
|  | SVM | Epidemiological Data | 1 |
|  | RF | Biological Data | 1 |
|  | KNN | Environmental Data | 1 |
|  | DT | Environmental Data | 1 |
|  | RNN | Environmental Data | 1 |
|  | Boosting | Images Or Videos | 1 |
|  | CNN | Images Or Videos | 1 |
|  | ANN | Socio Economic Data | 1 |
|  | LoR | Socio Economic Data | 1 |
|  | KNN | Entomological Data | 1 |
|  | ANN | Entomological Data | 1 |
|  | LoR | Entomological Data | 1 |
|  | DT | Entomological Data | 1 |
|  | RNN | Entomological Data | 1 |
|  | KNN | Animal Data | 1 |
|  | ANN | Animal Data | 1 |
|  | SVM | Animal Data | 1 |
|  | CNN | Animal Data | 1 |
|  | LoR | Animal Data | 1 |
|  | Clustering | Animal Data | 1 |
|  | CNN | Spectrocopy | 1 |
|  | LoR | Demographic Data | 1 |

**Table S3** Representative examples of borderline classification decisions across epidemiological stages

| **Study** | **Method** | **Prediction target** | **Assigned stage** | **Classification rationale** |
| --- | --- | --- | --- | --- |
| Moua et al. [1] | MaxEnt + environmental data | Spatial distribution of *An. darlingi* in French Guiana | Animal reservoir/vector | Prediction target is vector habitat suitability; analysis remains within the vector system with no reference to human infection. |
| Davis et al. [2] | RF + physiological telemetry | Pathogen exposure status in individual non-human primates | Animal reservoir/vector | Individual-level pathogen detection within a known host species; no inference about cross-species transmission to humans. |
| Plowright et al. [3] | Generalized boosted regression + species traits | Bat species in India likely to host Nipah virus | Animal-human interface | Aims to discover novel reservoir species to guide surveillance for preventing human spillover; prediction target identifies potential sources of human infection. |
| Evans et al. [4] | Ecological network model + vector-virus traits | Unknown Zika virus vector species | Animal-human interface | Predicts novel vector species that could mediate transmission to humans; the analytical goal explicitly bridges the animal and human systems. |
| Sallam et al. [5] | MaxEnt + biophysical and socioeconomic data | Vector-host contact ratios for WNV in New Orleans | Animal-human interface | Outcome variable is the contact ratio between mosquito vectors and human hosts, directly modeling the animal-human transmission link. |
| Keyel et al. [6] | RF + climate and hydrological data | WNV infection rates in mosquitoes and human case counts | Animal reservoir/vector and animal-human interface | Contains two research objectives: mosquito infection rate (animal stage) and human case counts linked to vector exposure (interface stage); assigned to both stages. |
| Basinski et al. [7] | Multi-layer BRT + environmental and serosurvey data | Reservoir distribution and Lassa virus spillover rate | Animal reservoir/vector and animal-human interface | First layer predicts reservoir host distribution (animal stage); second layer estimates spillover to humans using human serosurvey data (interface stage); assigned to both stages. |

**References**

1. Y. Moua, E. Roux, R. Girod, et al. “Distribution of the Habitat Suitability of the Main Malaria Vector in French Guiana Using Maximum Entropy Modeling.” *Journal of Medical Entomology* 54, no. 3 (2016): 606-621.

2. S. Davis, L. Milechin, T. Patel, et al. “Detecting Pathogen Exposure During the Non-symptomatic Incubation Period Using Physiological Data: Proof of Concept in Non-human Primates.” *Frontiers in Physiology* 12, (2021): 691074.

3. R. K. Plowright, D. J. Becker, D. E. Crowley, et al. “Prioritizing surveillance of Nipah virus in India.” *PLOS Neglected Tropical Diseases* 13, no. 6 (2019): e0007393.

4. M. V. Evans, T. A. Dallas, B. A. Han, C. C. Murdock, & J. M. Drake. “Data-driven identification of potential Zika virus vectors.” *Elife* 6, (2017): e22053.

5. M. F. Sallam, S. R. Michaels, C. Riegel, et al. “Spatio-Temporal Distribution of Vector-Host Contact (VHC) Ratios and Ecological Niche Modeling of the West Nile Virus Mosquito Vector, Culex quinquefasciatus, in the City of New Orleans, LA, USA.” *International Journal of Environmental Research and Public Health* 14, no. 8 (2017): 892.

6. A. C. Keyel, O. Elison Timm, P. B. Backenson, et al. “Seasonal temperatures and hydrological conditions improve the prediction of West Nile virus infection rates in Culex mosquitoes and human case counts in New York and Connecticut.” *PloS One* 14, no. 6 (2019): e0217854.

7. A. J. Basinski, E. Fichet-Calvet, A. R. Sjodin, et al. “Bridging the gap: Using reservoir ecology and human serosurveys to estimate Lassa virus spillover in West Africa.” *PLOS Computational Biology* 17, no. 3 (2021): e1008811.
